# Factors affecting the efficiency of equine embryo transfer (EET) in polo mares under subtropical conditions of Pakistan

**DOI:** 10.1371/journal.pone.0298066

**Published:** 2024-02-12

**Authors:** Khalid Mahmood, Aijaz Ali Channa, Aamir Ghafoor, Amjad Riaz

**Affiliations:** 1 Department of Theriogenology, University of Veterinary and Animal Sciences, Lahore, Punjab, Pakistan; 2 University Diagnostic Lab (UDL) at Institute of Microbiology, University of Veterinary and Animal Sciences, Lahore, Punjab, Pakistan; Western Michigan University School of Medicine: Western Michigan University Homer Stryker MD School of Medicine, UNITED STATES

## Abstract

Equine embryo transfer (EET) is a prominent technology in the equine breeding industry, and its efficacy is affected by a number of factors. The current study aimed to determine the effects of the breed of donor/recipient mares, estrus/ovulation induction treatment, cooled transportation of embryos, and synchrony between donor and recipient mares on the efficiency of the EET under subtropical conditions of Pakistan. A total of eighty-four (n = 84) Polo-playing donor mares (Argentino-polo = 41 and Anglo-Arab = 43) and seventy (n = 70) recipient mares (light breed = 26 and heavy breed = 44) were used for EET. The donor mares exhibiting natural estrus (n = 28) were detected by teaser a stallion, and corpus luteum (CL) having mares (n = 56) were treated with prostaglandin (150 μg of Cloprostenol) for estrus induction. The mares’ follicular growth was monitored through ultrasonography until the dominant follicle’s size reached 35 mm or more with a moderate to obvious uterine edema score. Afterward, the mares were treated either with GnRH, i.e., 50 μg of Lecirelin acetate (n = 41) or Ovusyn, i.e., 1500 IU hCG (n = 43). Insemination with chilled semen was performed 24 hours later. The embryos were collected non-surgically, 7 or 8 days after ovulation, from the donor mares. The collected embryos were transferred into the well-synchronized recipient mares as fresh (n = 44) or chilled (n = 26) embryos. The pregnancy after ET was checked through ultrasonography. Statistical analysis revealed that the embryo recovery rate (ERR) remained significantly higher (P<0.05) for the Prostaglandin (PG) treated group of donors as compared to the natural heat group of donors. The breed of donor mares, type of ovulatory treatment given, and day of embryo collection did not significantly (P>0.05) affect the ERR. There was no significant effect of the type (fresh vs chilled), classification, and stage of development of embryo on pregnancy outcomes (P>0.05). ET pregnancy rate was significantly affected by the breed of recipient mares and ovulation synchrony between donor and recipient mares (P<0.05). In conclusion, under the subtropical conditions of Pakistan, PG-based estrus induction of donor mares, breed of recipient mares, and ovulation synchrony between the donor and recipient mares had a substantial effect on the efficiency of EET.

## Introduction

Equestrian sports are a massive industry across the globe and have a socio-economic and cultural role in Pakistan as well. Reproductive biotechnologies like semen processing, reproductive ultrasonography, AI, and equine embryo transfer (EET) can help to produce high-quality equines in a shorter time [1, 2]. However, the use of reproductive biotechnology among horse breeders in developing countries like Pakistan is very limited. The possible reasons for not adopting these equine reproductive biotechnologies in developing countries are the lack of infrastructure, technical human resources, and breed regulations[3, 4].

Equine embryo transfer began in the 1970s with modest success by Japanese and Cambridge (England) organizations. Later, non-surgical EET with acceptable conception rates was established, but its widespread use was limited compared to other domestic species [5]. The two major barriers to the widespread use of EET in the 1970s were breed registration authorities’ non-acceptance of AI/ET in mares [6] and poor superovulation results [7, 8]. Cryopreservation of stallion semen and mare embryos is also a big challenge [9–11]. Advancements in chilled semen and cooled embryo transport have been a great breakthrough in equine reproductive biotechnologies. Now, it is possible to transport equine semen/embryos instead of transporting a mare or stallion without compromising the fertility results [9, 12, 13]. Cooled transport of mare embryos with satisfactory results has given momentum to EET [9] as it has eliminated the need to keep recipient and donor mares at the same stable. Large recipient mare centers are being maintained in some countries like Argentina/Brazil to facilitate donor mare owners [14].

EET is very popular in sports mares nowadays, as it enables breeders to produce foal from their sports mares without affecting their athletic performance [14–16]. The success of EET is affected by many intrinsic (fertility of stallion, donor and recipient mares) and extrinsic (climatic conditions, professional expertise of the veterinarian, Housing and Management of mares, etc.) factors [17–20]. The most important and expensive limiting factor in equine embryo transfer programs is the availability of suitable recipient mares [21]. Synchronization between the donor and recipient mare is a very important factor for the success of EET. Maintaining a group of recipients for ET is the most expensive segment of the equine embryo transfer industry. To reduce the costs, the EET industry has been divided into three segments: donor mare owners, assisted reproductive laboratories, and recipient centers. The embryos collected from the donor mares are shipped as "cooled embryos" to the recipient centers and transferred into the synchronized recipient mares [21–24]. The methods of shipment and preservation of equine embryos allow mare owners in remote locations to access sophisticated assisted reproductive technologies [25–27].

The embryos are collected from donor mares on day 7 or 8 after ovulation and transferred into the recipient mares between day 5 to day-8 post-ovulation. It is always desirous to have a recipient mare ovulated 1 or 2 days later than donor mares [21]. Different hormonal products have a definite role in EET programs. GnRH analogs, hCG, Prostaglandin analogs, Oxytocin, and Progesterone (P4) supplementation are the primary hormones for equine breeding management and EET Programs [24, 25, 28, 29]. GnRH analogs and hCG are used for ovulation induction, and prostaglandin is used for estrus induction by shortening the diestrus phase by luteolysis [30].

The reported pregnancy rate in recipient mares ranges between 65 to 85% on day-14 after ovulation [31]. The factors affecting EET programs include donor factors, embryo factors, recipient factors, male origin factors, and technical factors [32]. Donor mares’ fertility is affected by age, breed, intrinsic fertility, parity, sports activity, nutritional status, lactational status, and other reproductive pathologies [33]. Recipient factors include timing of ovulation (i.e., synchrony between the donor and recipient mares), intrinsic fertility of recipient mare, length of preceding estrus, nutritional and health status of a recipient mare, and circulating P4 level at the time of embryo transfer [21, 23, 34–36]. Embryo factors include the quality of the embryo, age and size of the embryo, and technical factors include the type of embryo (fresh, chilled, or frozen), the efficiency of a technician during collection, lab handling and transfer, and ET technique [8, 34, 37]. The male origin factors are translated into semen quality used for insemination as chilled or frozen semen. A good quality stallion semen increases the chances of embryo recovery rate from donor mares [38, 39].

In Pakistan, one can find equines of exceptional quality, and a considerable number of Anglo-Arab and Argentino-polo mares are affiliated with several polo clubs that are officially recognized by the Pakistan Polo Association (PPA). The term "Argentino-polo mares" refers to polo horses raised in Argentina for polo competition [40]. Argentina’s commercial EET centres are world-renowned since it is the leader in the equine ET business, where EET creates polo ponies [9, 40, 41]. Indigenously, Anglo-Arab horses have proved themselves the best as polo ponies in Pakistan. Anglo-Arab mares are a cross of Arab and Thoroughbred breeds of horses. EET can help to produce offspring from these polo-playing mares without affecting their sports career. Previously, there were no reports about the use of EET / factors affecting the efficiency of EET in Pakistan. The objective of the current study was to determine the effects of the breed of donor/recipient mares, estrus/ovulation induction treatment, cooled transportation of mare embryos, and synchrony between donor and recipient mares on efficiency EET in polo mares under subtropical conditions of Pakistan.

## Materials and methods

### Donor/Recipient mares

A total of eighty-four (n = 84) Polo-playing mares (Argentino-polo = 41 and Anglo-Arab = 43) were used as embryo donors. The donor mares were 4 to 10 years old and in good body condition. Seventy (n = 70) mares were used as recipient mares for embryo transfer. The recipient mares were 4 to 5 years old and in good body condition. Twenty-six of the recipient mares (n_1_ = 26) were of light breed (Thoroughbred, Arab, or their crosses), and forty-four (n_2_ = 44) of the recipient mares were of heavy breed (Percheron). All the donor/recipient mares were maiden mares and regular cyclic during the breeding season.

#### Ethical approval

All the experiments were approved by the ethical review committee of the University of Veterinary and Animal Sciences Lahore (DR/775 dated 20 Feb 2021) following ARRIVE guidelines, and informed consent was obtained from the mare owners. No animal was harmed or euthanized during the study.

#### Nutrition and housing management of mares

The study was conducted in Punjab (31.1704° N and 72.7097° E), Pakistan, during the equine breeding season (March to September). Balanced nutrition (consisting of oats/lucern hay, green fodder, grain-based concentrate, and vegetable oil) and fresh drinking water were provided to all the mares [42]. The donor mares were housed in individual stabling boxes. The recipient mares were kept in open paddocks having housing sheds installed. The mares were dewormed/vaccinated regularly. Good sanitary and hygienic animal housing conditions, feeding mangers, and water troughs were ensured regularly. Electric fans were also installed in the animal boxes if required due to summer stress.

#### Breeding methods

The donor mares were inseminated with chilled semen. Semen from four (n = 4) polo-playing stallions of Argentino-polo / Anglo-Arab bloodline was used for donor mares. All these stallions were selected as per their previous fertility records. Breeding soundness [43] was also evaluated before the execution of the study.

#### Semen collection and processing

The semen to be used for AI was collected in Colorada equine AV, i.e., Artificial Vagina (Ref. 11230/0000 Minitub GmbH, Germany), as per the manufacturer’s guidelines. The collected semen samples were evaluated for concentration and motility using a photometer calibrated for equines (SDM 1, ref. 12300/0101 Minitub GmbH, Germany) and a phase-contrast microscope (MBL 2000, Kruss optronic GmbH, Germany).

To produce the chilled semen doses, a skim milk-based semen extender with antibiotics (Equiplus, ref:13570/0210 Minitub GmbH, Germany) was used per manufacturer instructions. The minimum number of motile sperm per insemination dose was 600 million [44]. The inseminated volume ranged between 10–30 ml. After the dilution, the semen was not maintained in warm conditions. It was kept in the semen transport box (REF.: 17229/0002 GmbH Minitube, Germany) or equitainer (Ref:17220/0015 GmbH Minitube, Germany) without ice packs/frozen cans for about 20 minutes, and then ice packs/frozen cans were kept in place.

#### Artificial insemination of the donor mares

Non-surgical AI of the donor mares was performed at a designated time. The required volume of semen dose was filled in the sterile syringe and connected to the mare AI canula or pipette [12, 13]. Briefly speaking, the genitalia of the mare was cleaned. A sterile sleeve was worn, and an AI cannula (held in a cupped hand) was taken into the vagina. Non-spermicidal jelly was applied first before starting AI. The mare AI canula or pipette was gently passed through the cervix. The semen was slowly deposited into the uterus, and the AI cannula was withdrawn.

#### Ultrasonography

Reproductive ultrasonography was performed using a Draminiki iScan2 multi-ultrasound scanner with a linear rectal probe at 7.0 MHz. The mares were scanned as described in the relevant study sections. Briefly speaking, the mares in estrus were scanned every 12 hours until one-day post ovulation [45, 46]. The pregnancy of the recipient mare (after ET) was checked on day 14, day 28, and day 45 of embryonic/fetal age through ultrasonography [47]. A few pictures of ultrasonic scanning are shown in S3 and S4 Figs.

#### Estrus induction

The donor/recipient mares having corpus luteum (CL) were treated with prostaglandin (Dalmazin, Fatro-Itlay). The mares with a CL were intramuscularly treated with 2 ml of Dalmazin (150 μg of Cloprostenol). The mares were then scanned 4–5 days after Dalmazin treatment, and ultrasonographic scanning continued throughout the estrus till one day post-ovulation. The mares coming into estrus after spontaneous luteolysis were considered as mares with natural heat [48].

#### Ovulation induction

Once the dominant follicle’s size reached 35 mm or more, along with moderate to obvious endometrial edema, the donor mares were treated with GnRH (Dalmarelin, Fatro-Itlay) or hCG (Ovusyn, Zoetis-Argentina) as per manufacturer recommendation for ovulation induction. Briefly speaking 2ml of Dalmerilin (50 μg of Lecirelin acetate) was administered intravenously to GnRH group mares (n = 41), and 3 ml of Ovusyn (1500 IU hCG) was administered intravenously to hCG group mares (n = 43). The ovulation was monitored after every 12 hours of this treatment. Only GnRH (Dalmarelin, Fatro-Itlay) was administered to all the recipient mares for ovulation induction.

### Experimental design

In this study, a total of 84 Polo-playing mares (Argentino-polo = 41 and Anglo-Arab = 43) were used as embryo donors and 70 recipient mares (light breed = 26 and heavy breed = 44) were used for embryo transfer. The collected embryos were then transferred into the recipient mares. The effects of the breed donor mares, estrus/ovulation induction treatment, and day of embryo collection on equine embryo production were studied.

The mares coming into heat naturally (n_1_ = 28) were ascertained through a teaser stallion. The mares having corpus luteum (CL) were treated with Prostaglandin (PGF2α) hormone (n_2_ = 56) for estrus induction. The mares in estrus were monitored through ultrasonography for follicular growth and uterine edema score. The mares were treated with GnRH or hCG hormone for ovulation induction on attaining a size of 35 mm or above, along with a moderate to obvious uterine edema. Mares were inseminated using chilled semen of Polo breeding stallions, 24 hours after ovulation induction. The ovulation was monitored every 12 hours post-AI. The mare was re-inseminated if ovulation did not occur in the donor mares up to 48 hours post-AI. The embryos were collected non-surgically from the inseminated donor mares seven to eight days post-ovulation [8, 49]. The day of ovulation was considered as day "0 ".

The collected embryos were graded according to the stage of development, shape, and cellular content [50]. This evaluation was based on certain factors such as size/stage of development (e.g., morula, blastocyst) and shape of embryo, blastomere condition, uniformity/integrity of the zona pellucida and the general appearance of the embryo (including any symptoms of abnormalities or degeneration). The embryos were either transferred as fresh or chilled. Embryos were transferred non-surgically [8, 49]. The experimental timeline is shown in S1 Fig.

#### Embryo collection and processing

The embryos were collected non-surgically [49] using a Silicon embryo flushing catheter for mares (CH-32, ref. 19909/0032, Minitub GmbH, Germany) attached with a Y-junction with a high flow connector spike port (19982/0202 Mintube USA). The flushed media (ringer lactate) passed through the Emsafe system for embryo collection (Ref.19010/600 Minitub GmbH, Germany).

The embryo was viewed through the naked eye in the embryo collection filter after every liter of the flushing media. The next liter of the flushing media was used if the embryo was not seen. A maximum of three liters of flushing media was used, and then the embryo filter was detached from the flushing assembly. It was taken into the lab, and the embryo was searched under a stereo-zoom microscope. The searched embryo was picked using a 0.5ml straw fixed in an A mode micro-pipettor for embryo handling (Ref.19022/000, Minitub GmbH, Germany). The collected embryos were washed in embryo-holding media (Equihold ref. 19982/6250 Lot# 80181105301, Minitub GmbH, Germany) multiple times before placing them in the small petri dish having holding media [51, 52]. This equine embryo-holding media was pre-warmed at 35 °C on a laboratory heating plate. The collected embryos were graded according to the stage of development, shape, and cellular content [6, 50]. As S5 Fig, a few pictures of mare embryos have been shared. The chilling or cooling of the embryo was performed in embryo-holding media (Equihold ref. 19982/6250 Lot# 80181105301, Minitub GmbH, Germany) as per manufacturer guidelines, and embryos were stored in Equitainer-I [53]. The cooled stored embryos were then transferred into the recipient mares within 24 hours after storage.

#### Embryo transfer technique

Embryos were transferred non-surgically [8, 49] using an equine embryo transfer ET cannula with side openings (Ref. 19290/1060 Minitub GmbH, Germany). Briefly speaking, the tail of the recipient was wrapped and tied upwards. The feces in the rectum were removed, and the perineum area was washed with non-residual soap. Afterward, this area was washed with fresh water and dried with a paper towel. The organic debris in the vestibule was removed by a moist cotton swab. The embryo was loaded into the 0.5 ml straw and fixed on a stiff stylet (Ref. 19290/1049 Minitub GmbH, Germany) before inserting it into the ET cannula. A hygienic sheath (Ref. 19271/0080 Minitub GmbH, Germany) was also pulled on the ET cannula. The tip of the ET cannula was held in a cupped hand having sterile glove. A non-spermicidal gel was applied on the back side of the hand, and it was gently inserted into the vagina. The tip of the ET cannula was guided into the cervix with the help of the index finger and thumb. The hygienic sheath was pulled back once the ET cannula entered the cervix. The ET cannula was gently pushed through the cervix until it reached the body of the uterus. The embryo was gently placed in the uterus by pushing the stiff stylet [6].

#### Management of recipient mares

All the mares were selected based on previous reproductive history/breeding soundness exam. The cyclic recipient mares (having CL) were treated with PGF2α, so that they may come into estrus (one or two days later than donor mares). Similarly, a mare or group of mares coming into estrus after spontaneous luteolysis was considered in natural estrus. The mares in estrus were given GnRH treatment (after attaining 35 mm or more sized dominant follicle along with moderate uterine edema score) for ovulation induction, preferably two days later than donor mares.

The recipient mares were followed by reproductive ultrasonography after every 12 hours till one day post-ovulation. The recipient’s quality (to evaluate its suitability for EET) was determined a day before embryo transfer through reproductive ultrasonography. The recipient mares were graded into good, acceptable, and marginally acceptable based on the quality of CL, endometrial edema score, tone of uterus/cervix, and general body condition [6, 36, 54]. The recipient mares with optimal characteristics, including high-quality Corpus Luteum (CL), no uterine edema, good cervical/uterine tone, and no history of reproductive issues, were classified as ’good recipients.’ Those with minor deviations from these ideal conditions, such as slightly poor cervical/uterine tone or minor variations in CL quality, were considered ’acceptable.’ Lastly, mares with more pronounced issues, like poor uterine/cervical tone, mild uterine edema, or a resolved history of health or reproductive problems, were labeled as ’marginally acceptable.‴ The embryos were transferred into the recipient mares ovulating the same day to three days later (0 to -3 days) than the donor mares.

#### Study variables

The effects of the breed of donor mare, estrus/ovulation induction treatment, and day of embryo collection (after ovulation) on ovulation rate, embryo recovery rate (ERR), and quality/classification of embryos were monitored in donor mares. The effects of the breed and quality of recipient mares, synchrony between the recipient and donor mare, quality /classification of the embryo, and type of embryo (fresh vs chilled) on pregnancy rate (PR) after ET were evaluated in recipient mares.

#### Statistical analysis

Fisher’s exact test with a 95% confidence interval was used to investigate the effects of the independent variables (breed of donor mare, type of ovulatory / estrus induction treatment, and day of embryo collection) on dependent variables of donor mares (ovulation rate/embryo recovery rate (ERR) and quality/classification of embryos) and effects of the breed of recipient mares on quality of recipient mares. To study the effects of the breed/quality of a recipient mare, embryo-related factors (type, classification, and quality), PG-treatment to recipient mares, and synchrony between donor and recipient mares on pregnancy rate (PR), a binary logistic regression test with a 95% confidence was conducted. All statistical analysis was performed in SPSS 29.0.

## Results

### Factors affecting equine embryo production

#### Effect of the breed of donor mares on embryo recovery rate

The successful embryo recovery percentages between Argentino-polo and Anglo-Arab mares are shown in Table 1. In total, 72 embryos were collected from 84 collections. Among Argentino-polo donor mares (n = 41), 36 embryos were collected. Among Anglo-Arab donor mares (n = 43), 36 embryos were collected. Both breeds yielded similar results, with no statistically significant difference (P > 0.05). The details are summarized in Table 1.

**Table 1:** Effect of the breed of donor mare on embryo production parameters.

Serial	Parameter	Breed of Donor Mare		*P*-Value
		Argentino-Polo (n = 41)	Anglo-Arab (n = 43)	
1	**Embryo Recovery Rate**			1.000
	Positive Collection	68.3% (28/41)	69.8% (30/43)	
	Negative Collection	31.7% (13/41)	30.2% (13/43)	
2	**Number of embryos per collection**			0.731
	1-embryo	48.8% (20/41)	55.8% (24/43)	
	2-embryos	19.5% (08/41)	14.0% (06/43)	
	No-embryos	31.7% (13/41)	30.2% (13/43)	
3	**Number of ovulations**			0.273
	Single Ovulation	75.6% (31/41)	86.0% (37/43)	
	Double Ovulation	24.4% (10/41)	14.0% (06/43)	
4	**Classification of embryos**			0.909
	Morula	5.7% (02/37)	5.4% (02/35)	
	Blastocyst	77.1% (29/37)	75.7% (28/35)	
	Expanded blastocyst	17.1% (06/37)	18.9% (07/35)	
5	**Quality of embryo**			0.097
	Excellent	94.3% (35/37)	81.3% (28/35)	
	Good	05.7% (2/37)	16.2% (06/35)	
	Fair	0% (0/35)	01.4% (01/35)	

Values in bold are significant at P < 0.05.

#### Effect of the breed of donor mares on multiple ovulations

Although no superovulation treatment was given in this study, however, 19.04% (16/84) of the donor mares showed double ovulation. The percentage of double ovulations in Argentino-polo mares was 24.4% (10/41), and the percentage of double ovulation in Anglo-Arab mares was 14% (6/43). The difference was statistically non-significant at a 95% confidence interval. The summary of the results is shown in Table 1.

#### Effect of breed on the classification of collected embryos

There was no statistically significant effect of the breed of the donor mare on the classification of collected embryos. The maximum percentage, i.e., 79.16% (57/72) of collected embryos, was blastocyst. The percentages for morulas and expanded blastocysts were 5.5% (4/72) and 21.6% (13/60), respectively. The percentages for morulas and blastocysts were higher for Argentino-polo mares, while the percentage of expanded blastocysts was higher for Anglo-Arab mares. All these results were statistically non-significant. The summary of the results is shown in Table 1.

#### Effect of the breed of donor mares on the quality of the embryo

The quality of the collected embryos was not affected by the breed of the donor mare. Overall, 87.5% (63/72) of embryos were of excellent quality. The percentage of excellent-quality embryos was 94.3% (35/37) for Argentino-polo mares and 81.3% (28/35) for Anglo-Arab mares. The percentage of good-quality embryos was 05.7% (02/37) for Argentino-polo mares and 16.2% (06/35) for Anglo-Arab mares. No fair or poor embryo was collected from Argentino-polo mares; only 01.4% (01/35) of embryos collected from Anglo-Arab mares were of poor quality. All these differences were statistically non-significant. Overall, only 1.3% (1/72) of embryos were of poor quality, and the breed of the donor mare showed no impact on the quality of the embryos collected during this study. The results have been summarized in Table 1.

#### Effect of ovulation induction treatment on embryo recovery in donor mares

Cumulatively, there was no statistically significant effect (p>0.05) of ovulatory treatment given to the donor mares on ERR per flushing. The percentage of 2-embryos recovery per flushing remained higher for the ovusyn group as compared to the GnRH group, as shown in Table 2. The percentage of double ovulations in the hCG (Ovusyn) group remained higher as compared to the GnRH group. This may be a reason for the higher percentage of two embryo recoveries per flushing for the hCG group. However, all the differences were statistically non-significant at a 95% confidence interval. All the mares (except one) ovulated within 48 hours after ovulation induction treatment. The one mare that did not ovulate within 48 hours was re-inseminated and ovulated within 72 hours after ovulation induction treatment. The results have been summarized in Table 2.

**Table 2:** Effect of ovulatory treatment given to donor mares on embryo production parameters.

Serial	Parameter	Ovulatory Treatment		*P-*value
		GnRH (n = 41)	hCG (n = 43)	
1	**Embryo Recovery Rate**			0.158
	Positive Collection	61.0% (25/41)	69.6% (33/43)	
	Negative Collection	39.0% (16/41)	30.4%(10/43)	
2	**Number of embryos per collection**			0.058
	1-embryo	53.7% (22/41)	51.2% (22/43)	
	2-embryos	07.3% (3/41)	25.6% (11/43)	
	No-embryos	39.0% (16/41)	23.3% (10/43)	
3	**Number of ovulations**			0.051
	Single Ovulation	90.2% (37/41)	72.1% (31/43)	
	Double Ovulation	9.8% (04/41)	27.9% (12/43)	

Values in bold are significant at P < 0.05.

#### Effect of Prostaglandin (PG) treatment on embryo recovery from donor mares

The donor mares having Corpus luteum (n = 56) were given PG-treatment to induce estrus. The percentage of embryo recovery from PG-treated donor mares was significantly higher, i.e., 76.8% (43/56) compared to 53.6% (15/28) of the non-PG group. The percentage of double ovulations and two embryos per flushing was also higher for PG-treated donor mares. However, only the percentage of double ovulations for PG-treated donor mares was significantly higher, and not the two embryos per flushing. The results have been summarized in Table 3.

**Table 3:** Effect of the type of estrus on embryo production parameters.

Serial	Parameter	Type of estrus		*P*-Value
		PG-induced (n = 56)	Natural (n = 28)	
1	**Embryo Recovery Rate**			**0.045**
	Positive Collection	76.8% (43/56)	53.6% (15/28)	
	Negative Collection	23.2% (13/50)	46.4% (13/28)	
2	**Number of Ovulations**			0.075
	Single Ovulation	75% (42/56)	92.85% (26/28)	
	Double Ovulation	25% (14/56)	07.14% (2/28)	
3	**Number of embryos per collection**			0.104
	1-embryo	57.1% (32/56)	46.4% (13/28)	
	2-embryos	19.6% (11/56)	10.7% (03/28)	
	No-embryos	23.2% (13/56)	42.9% (12/28)	

Values in bold are significant at P < 0.05.

#### Effect of the day of embryo collection on embryo recovery rate

Out of 84 embryo flushings, maximum flushings, i.e., 83.33% (70/84), were made on day-8 after ovulation, and 16.67% (14/84) flushings were made on day-7 after ovulation. The percentage of successful embryo recovery from donor mares was 64.3% (9/14) and 70.0% (49/70) on day-7 and day-8 after ovulation. All the differences were statistically non-significant at a 95% confidence interval except for the classification of embryos. The summary of the results has been presented in Table 4.

**Table 4:** Effect of the day of flushing on embryo production parameters.

Serial	Parameter	Day of Embryo Collection		*P*-Value
		Day-7 (n = 14)	Day-8 (n = 70)	
1	**Embryo Recovery Rate**			0.754
	Positive Collection	64.3% (9/14)	70.0% (49/70)	
	Negative Collection	35.7% (5/14)	30.0% (21/70)	
2	**Number of embryos per collection**			0.190
	1-embryo	64.3% (9/14)	50.0% (35/70)	
	2-embryos	0% (0/14)	20.0% (14/70)	
	No-embryos	35.7% (5/14)	30.0% (21/70)	
3	**Classification of embryos**			**0.001**
	Morula	44.4% (4/9)	0% (0/72)	
	Blastocyst	55.6% (5/9)	79.4% (50/63)	
	Expanded blastocyst	0.0% (0/9)	20.6% (13/63)	
4	**Quality of embryo**			0.078
	Excellent	66.7% (6/9)	90.5% (57/63)	
	Good	33.3% (3/9)	11.1% (07/63)	
	Fair	0.0% (0/9)	1.6% (01/63)	

Values in bold are significant at P < 0.05.

### Factors affecting recipient performance

A logistic regression analysis was conducted to examine the association between various factors affecting EET pregnancy results and pregnancy outcome, and results are presented in Table 5. All the factors investigated contributed to the logistic regression model (χ2(10) = 28.525, p = 0.001). The Hosmer-Lemeshow test indicated a good fit for the model (χ2(6) = 9.673, p = 0.289).

**Table 5:** Factors affecting EET pregnancy results.

Parameter	N	Pregnancy outcome		P-value	Odds ratio (OR)	95% C.I. for EXP(B)
		Yes	No			
**Breed of Recipient**						
Light	26	73.1% (16/26)	26.9% (6/26)	**0.004**	Reference	
Heavy	44	29.5% (11/44)	70.5% (25/44)		0.120	0.028–0.508
**Prostaglandin Treatment**						
Yes	42	40.5% (17/42)	59.5% (25/42)	0.891	Reference	
No	28	53.6% (15/28)	46.4% (13/28)		0.906	0.223–3.676
**Type of embryo**						
Fresh	44	40.9% (13/44)	59.1% (19/44)	0.335	Reference	
Chilled	26	53.8% (14/26)	46.2% (12/26)		2.053	0.476–8.861
**Quality of recipient**						
Good	47	55.3% (21/47)	44.7% (14/47)	0.14	Reference	
Acceptable	23	26.1% (4/23)	73.9% (8/23)		0.337	0.080–1.427
**Synchronization (Days)**						
0	11	54.5% (6/11)	45.5% (5/11)	**0.034**	Reference	
-1	15	66.7% (10/15)	33.3% (5/15)	0.528	1.969	0.241–16.106
-2	21	52.4% (11/21)	47.6% (10/21)	0.986	1.016	0.188–5.49
-3	23	21.7% (5/23)	78.3% (18/23)	**0.013**	13.017	1.737–97.519
**Development Stage of embryo**						
Morula	4	50.0% (2/4)	50.0% (2/4)	0.57	Reference	
Blastocyst	51	43.1% (22/51)	56.9% (29/51)	0.944	0.883	0.027–28.871
Expanded blastocyst	15	53.3% (8/15)	46.7% (7/15)	0.363	2.364	0.37–15.1
**Quality of embryo**						
Excellent	62	46.8% (29/62)	53.2% (33/62)	0.51	Reference	
Good	8	37.5% (3/8)	62.5% (5/8)		0.478	0.053–4.279

Values in bold are significant at P < 0.05.

All the variables shown in the table are −2loglikelihood = 68.00; χ2(10) = 28.525, P = 0.001. Hosmer-Lemeshow statistics = 9.673 with 8 df, P = 0.289.

#### Effect of the breed of recipient mare on EET pregnancy results

In light-breed recipient mares, the EET pregnancy rate remained at 73.1% (16/26) compared to the 29.5% (11/44) of heavy-breed mares. The odds ratio for recipients classified as "Heavy" was 0.120 (95% CI: 0.028–0.508), indicating that heavy-breed mares have reduced odds of pregnancy compared to light-breed mares. Specifically, they have only 12% of the odds of becoming pregnant when compared with light-breed mares. This finding is statistically significant (p = 0.004), highlighting the importance of breed as a determinant factor in the success of EET. The results have been summarized in the Table 5.

### Effect of Prostaglandin treatment to recipient mares on EET pregnancy results

Prostaglandin (PG) treatment for estrus induction in the recipient mares did not demonstrate a significant association with pregnancy. The data indicate that mares not treated with prostaglandin had a higher pregnancy success rate (53.6%, 17/42) than those treated with Prostaglandin (40.5%, 17/42), though this difference was not statistically significant (p = 0.891). An OR of 0.906 (95% CI: 0.223–3.676) actually suggests that the odds of pregnancy in the Prostaglandin-treated group are slightly lower, the treated group has 9.4% lower odds of a pregnancy outcome compared to the non-treated group, although this is not a statistically significant difference (p = 0.891). The results have been summarized in Table 5 (S1 and S2 Datasets).

#### Effect of the breed on the quality of recipient mares

The recipient’s quality at the time of ET was affected by the breed of the recipient mare. In light breed mares, 80.8% (21/26) recipients were of good quality at the time of ET. While in heavy breed mares, 59.1% (26/44) recipients were of good quality. The percentage of acceptable recipients was higher for heavy breed recipients than for light breed recipients. Although these results were statistically non-significant (p = 0.072), but a significant impact was observed in EET pregnancy results due to breed differences of recipient mares. In S2 Fig, you can see a graph that shows how breed affects the quality of the recipient.

### Effect of the quality of recipient mares on pregnancy rate

Overall, the pregnancy rate for good-quality recipient mares (at the time of ET) was 55.3% (26/47) compared to 26.1% (06/23) in ’Acceptable’ quality mares. However, this association did not reach statistical significance (χ2(2) = 2.218, p = 0.330). The odds ratio for recipients classified as "acceptable" was 0.337 (95% CI: 0.080–1.427), indicating that mares of ’acceptable’ quality have approximately 66.3% lower odds of achieving a successful pregnancy outcome compared to those of ’Good’ quality. The results have been summarized in the Table 5.

#### Effect of the type of embryo on pregnancy rate

There has been no significant effect of the type of embryo (fresh vs chilled) in this study. The pregnancy rate for fresh and chilled embryos remained at 40.9% (13/44) and 53.8% (14/26), respectively, %), though this difference did not reach statistical significance (p = 0.335). The odds ratio for chilled embryos was 2.053 (95% CI: 0.476–8.861), suggesting that they have approximately twice the odds of a positive pregnancy outcome compared to fresh embryos. However, this observation is not statistically significant (p = 0.335), indicating that the finding could be due to chance and should be interpreted with caution. The summary of the results has been presented in Table 5.

#### Effect of Synchronization between the donor and recipient mares

Synchronization (of the day of ovulation) between the donor and recipient mares demonstrated a significant impact on pregnancy outcomes after ET. The maximum fertility rate was seen when recipient mares were one day behind donor mares (embryo age). Mares synchronized on day 0 had a pregnancy rate of 54.5% (6/11), which served as the reference category. A notable decline to a 21.7% (5/23) pregnancy rate was observed on day -3, with this decrease being statistically significant (P = 0.013). An OR of 1.969 (95% CI: 0.241–16.106) for synchronization at -1 day indicates that the odds of pregnancy are approximately double compared to synchronization at day 0, although this is not statistically significant (p = 0.528). The OR of 1.016 (95% CI: 0.188–5.49) for synchronization at -2 days indicates virtually no change in the odds of pregnancy compared to day 0 (p = 0.986). An OR of 13.017 (95% CI 1.737–97.519) for synchronization at -3 days reflects a substantial increase in the odds of pregnancy, indicating that the odds are 13 times lower compared to the reference time, which is statistically significant (p = 0.013). The summary of the results has been presented in Table 5.

### Effect of the embryo-related factors on the pregnancy rate of equine embryo transfer

#### Effect of the development stage of the embryo on pregnancy rate

Overall, there were 5.71% (4/70) morulas, 72.85% (51/70) blastocysts, and 21.42% (15/70) expanded blastocysts. The developmental stage of the embryo did not show a statistically significant effect on pregnancy rate, with morula, blastocyst, and expanded blastocyst stages yielding varying rates of pregnancy success (50.0%, 43.1%, and 53.3%, respectively) without reaching statistical significance. An OR of 0.883 (95% CI: 0.027–28.871), for the blastocyst stage indicates that there is a non-significant decrease in the odds of pregnancy compared to the morula stage, equating to about 11.7% less, although this is not statistically significant (p = 0.944). An OR of 2.364 (95% CI: 0.37–15.1) for the expanded blastocyst stage indicates that the odds of pregnancy are more than twice as high compared to the morula stage, though this finding is also not statistically significant (p = 0.363). The summary of the results has been presented in Table 5.

#### Effect of the quality of embryo on pregnancy rate

There was no significant effect of the embryo quality on the pregnancy rate of recipient mares. The maximum number of embryos, i.e., 98.6% (71/70), were of excellent or good quality. Only 1.4% (1/72) of embryos (not used for ET) were of fair quality. The quality of the embryo did not have a statistically significant impact on the pregnancy rate, with excellent quality embryos showing a pregnancy rate of 46.8% compared to 37.5% for good quality embryos (P = 0.478). An OR of 0.478 (95% CI: 0.053–4.279) for embryos of good quality indicates they have reduced odds of a positive pregnancy outcome than those embryos of excellent quality. However, this finding did not reach statistical significance (p = 0.51). The summary of the results has been presented in Table 5.

## Discussion

The equine embryo transfer (EET) industry is well-developed in many advanced countries, and the use of EET has increased manifold in the last few decades due to changes in breed regulations of major horse breeds in the USA and sports/polo horse industries of Brazil and Argentina [37]. The exact statistics of equine embryo transfer worldwide cannot be calculated due to under-reporting; however, the published embryo recovery rate in mares ranges between 50 to 75%, and expected chances of pregnancy after ET in well-synchronized recipient mares are about 75% [8]. Multiple external and internal factors can affect the efficiency of EET Programs [9, 17, 25, 54].

In our study, ERR was not significantly affected by the breed of donor mare (Argentino-polo vs Anglo-Arab), day of embryo flushing (7 or 8 after ovulation), and type of ovulatory treatment (GnRH vs hCG); however, PG-treated donor mares exhibited significantly higher ERR per flushing compared to mares coming into heat naturally.

No significant effect of the breed of donor mare (Argentino-polo vs Anglo-Arab) on ERR per flushing in our study is contrary to previous reports [39]. A retrospective study on donor mares of 10 breeding seasons has revealed that the ovulation rate and incidence of multiple ovulations were significantly affected by age, breed, and reproductive category [31].

In our study about the effects type of ovulatory treatment (GnRH vs hCG) administered to the donor mares, hCG-treated donor mares exhibited slightly better results for double ovulation and ERR. However, a study on performance mares of Argentina [15] has reported higher ERR with GnRH (Deslorelin, BioRelease Technologies, USA) than with hCG (Ovusyn, Suntex Buenos Aires, Argentina). In another study, combining hCG and deslorelin acetate to hasten ovulation could not change luteal development, progesterone concentration, or pregnancy outcome in recipient mares after ET [55]. Another study on American Quarter horse mares has concluded that ovulation induction rates in mares having uterine edema and ovarian follicle more than 35 mm were similar for new deslorelin acetate product SucroMate (89.9%) and hCG (82.8%) within 48 hours after drug administration [56]. A comparative study at the Equine Reproduction Laboratory, Colorado State University, on the clinical efficacy of compounded deslorelin and hCG at inducing timed ovulation in estrual mares with follicle sizes of 35 to 45 mm has shown no significant differences [57].

The results of our study about the effects of the day of flushing on ERR per flushing were in accordance with previous studies. Marinone et al. 2015 reported that the ERR was not affected by the day of flushing, i.e., 7, 8, or 9 days after ovulation [19]. Another Brazilian study of the equine ET program has reported no effect on the day of flushing (Day-7 to 10) [21]. However, the ET pregnancy rates varied significantly once the asynchrony between donor and recipient mares increased.

In our study use of exogenous PG in diestrus donor mares has exhibited significantly higher ERR per flushing than donor mares coming into estrus naturally. Using exogenous PG to induce luteolysis for estrus induction in diestrus mares is an effective tool for mare breeding management [58]. The possible reason for improved embryo recovery from PG-treated mares may be the better follow-up/coordination of estrus, follicular growth, and ovulation. This may also help to produce more embryos from a donor mare in a breeding season if she is treated with PG immediately after a flush [5, 58, 59]. A previous study in Argentino-polo mares has revealed that ERR in PG-treated donor mares increases as the interval between PG-treatment and upcoming ovulation increases; however, it does not affect subsequent PR after ET [60].

According to our study, ET pregnancy was significantly affected by the breed of recipient mare and synchrony between the donor and recipient mare; however, PG-treatment to recipient mares, type of embryo (fresh vs cooled-transported), and development stage of embryo exhibited no significant effects on the efficiency of EET.

In our study, the pregnancy results of heavy breed (draft mares) recipient mares remained significantly lower than the light breed recipient mares. According to our knowledge, no published reports compare two or more different breeds of recipient mares for pregnancy results after ET. However, the tone of the recipient mares’ uterus and cervix before the ET has been regarded as the most important factor in selecting a suitable recipient mare [36, 59]. In our study, a better uterine and cervical tone was constantly observed (through rectal palpation and ultrasonography) in light-breed recipient mares compared to heavy-breed mares. This may be a reason for better pregnancy results in light-breed recipient mares. Previously, it has been reported that ET pregnancy results in acceptable recipient mares are significantly better than marginally acceptable recipient mares [54]. However, according to the latest reports, such differences may be catered for by using appropriate hormonal preparations [2, 9]. Recent studies have reported successful pregnancies and foal births in recipient mules even [61, 62].

The results of our study about the synchronization between the donor and recipient mares revealed significant impacts on the pregnancy rate after an embryo transfer in the recipient mares. The recipient mares are the most critical and expensive limiting factor in EET programs [21]. In routine, embryos are collected from donor mares on day 7 or 8 after ovulation and transferred into the recipient mares between day 5 to day-8 post-ovulation [24]. Our study showed a significant difference in pregnancy rate between recipients having "-2 to 0-day synchronization" and "-3 day synchronization. Synchronization between the recipient and donor mares is critical in achieving higher pregnancy rates after EET [22, 24]. However, hormonal synchronization of recipient mares (especially with long-acting P4, PG, and E2) at any stage of the estrous cycle can help to achieve a satisfactory pregnancy rate in EET [24].

In our investigation, there was no significant influence of PG-based estrus induction on pregnancy outcomes in recipient mares. This result was in consistence with previous studies. Although a few studies have reported reduced pregnancy rates in recipient mares with the use of PG, but most of the studies revealed no differences in PG-synchronized and control mares [33].

Previous studies have shown significant effects of the stage of embryo development, size of the embryo, and quality of embryo on ET pregnancy in the recipient mares [54]. However, in our study, the ET pregnancy in recipient mares was not significantly affected by the embryo development stage and its quality. The possible reason behind this outcome was less variation in the quality and classification of collected embryos.

Our study has shown no significant effect on the type of embryo (fresh vs chilled). This finding is in accordance with previous studies [9, 32]. Cooled transport of mare embryos with satisfactory results has given momentum to EET as it has eliminated the need to keep recipient and donor mares at the same stable [14]. Recently, reported results on over 600 embryos shipped overnight to the ART laboratory for up to 19 hours showed no effect on fertility; however, shipment times of 20–24 hours significantly lowered the pregnancy rate [25]. The current study has revealed multiple factors affecting the efficiency of EET in polo mares under subtropical conditions in Pakistan and is expected to be used as a guideline for future EET programs in the country.

## Conclusion

It can be divulged from the present study that PGF2α-based estrus induction in polo-playing donor mares can improve ERR per flushing, and the use of well-synchronized light breed recipient mares can improve the efficiency of EET under subtropical conditions. Furthermore, cooled transport of equine embryos has no adverse effect on their fertility.

## Supporting information

S1 Dataset Donor raw sheet. (XLSX)

S2 Dataset Recipients raw data. (XLSX)

S1 Fig Study timeline. (JPG)

S2 Fig Breed effect on quality of recipient. (JPG)

S3 Fig Mare ovary scans. (PDF)

S4 Fig Mare uterine scans. (PDF)

S5 Fig Mare embryo pictures. (PDF)
