# Supplementary figures and images for: Factors affecting the efficiency of equine embryo transfer (EET) in polo mares under subtropical conditions of Pakistan
Source: PLoS One. 2024 Feb 12;19(2):e0298066. doi: 10.1371/journal.pone.0298066 (PMC10861068; doi:10.1371/journal.pone.0298066)

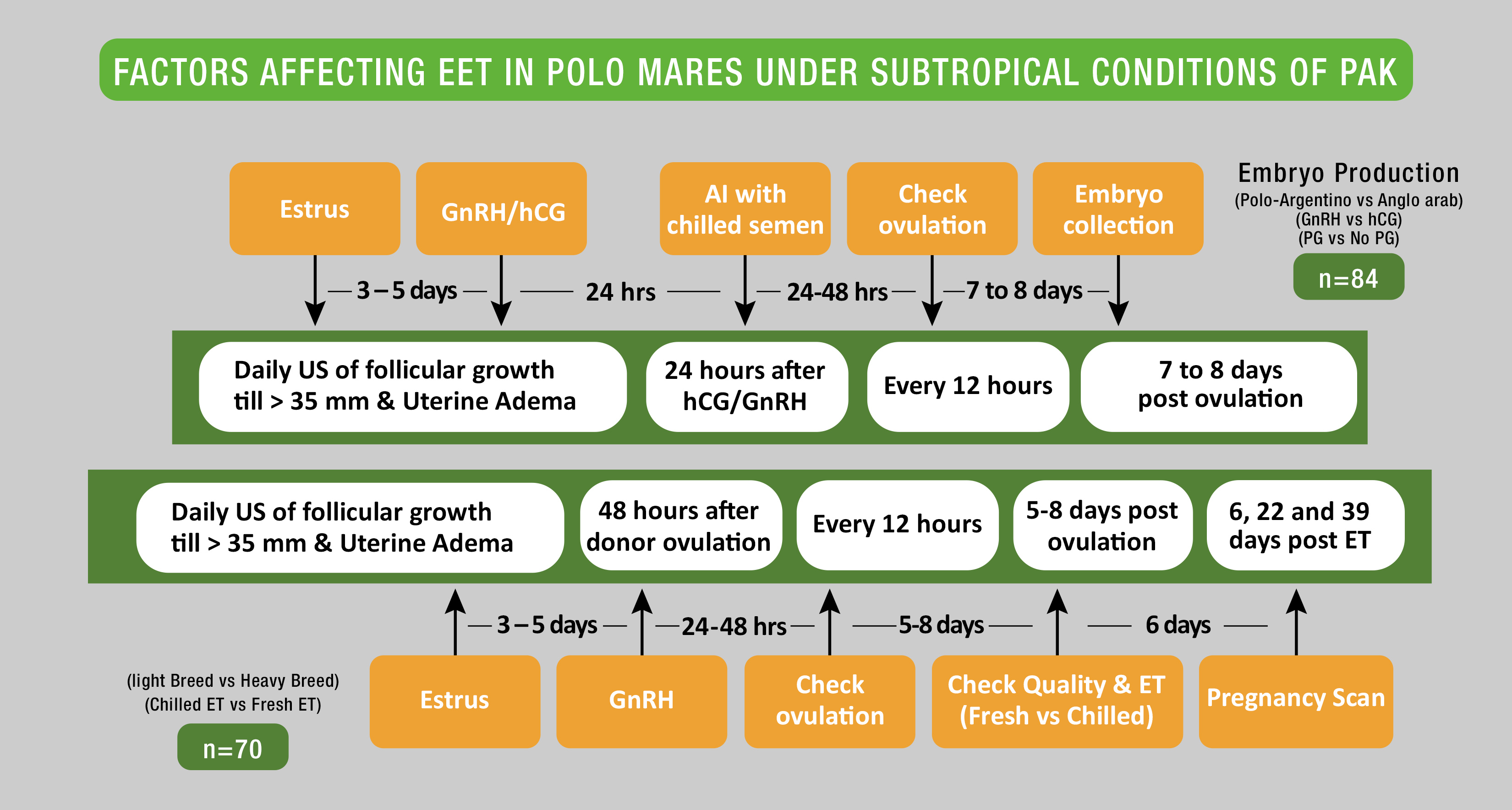

Supplement: S1 Fig — (JPG) [file pone.0298066.s003.jpg]

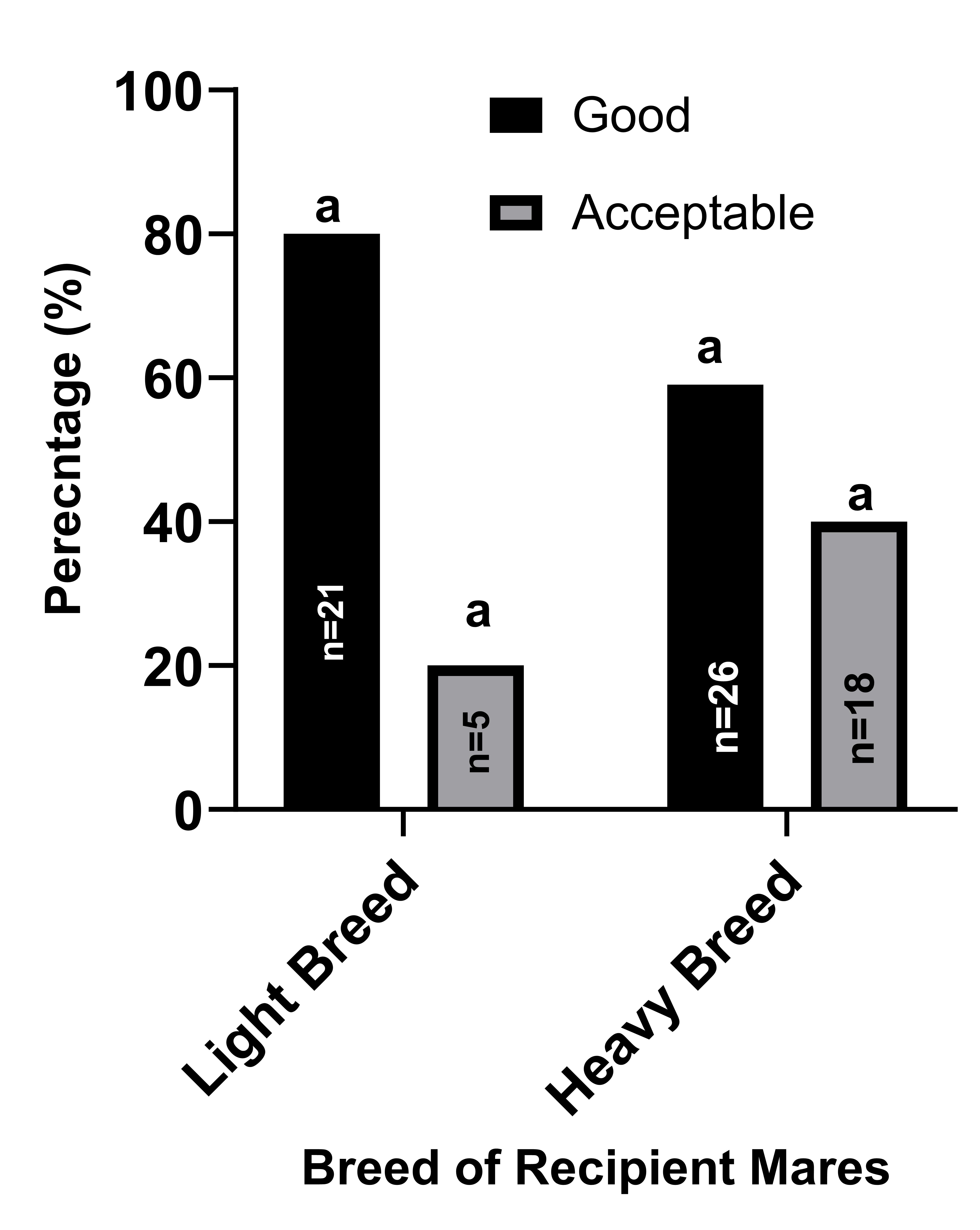

Supplement: S2 Fig — (JPG) [file pone.0298066.s004.jpg]
