# Supplementary material for: Factors affecting the efficiency of equine embryo transfer (EET) in polo mares under subtropical conditions of Pakistan
Source: PLoS One. 2024 Feb 12;19(2):e0298066. doi: 10.1371/journal.pone.0298066 (PMC10861068; doi:10.1371/journal.pone.0298066)
Supplement: S3 Fig — (PDF) [file pone.0298066.s005.pdf]

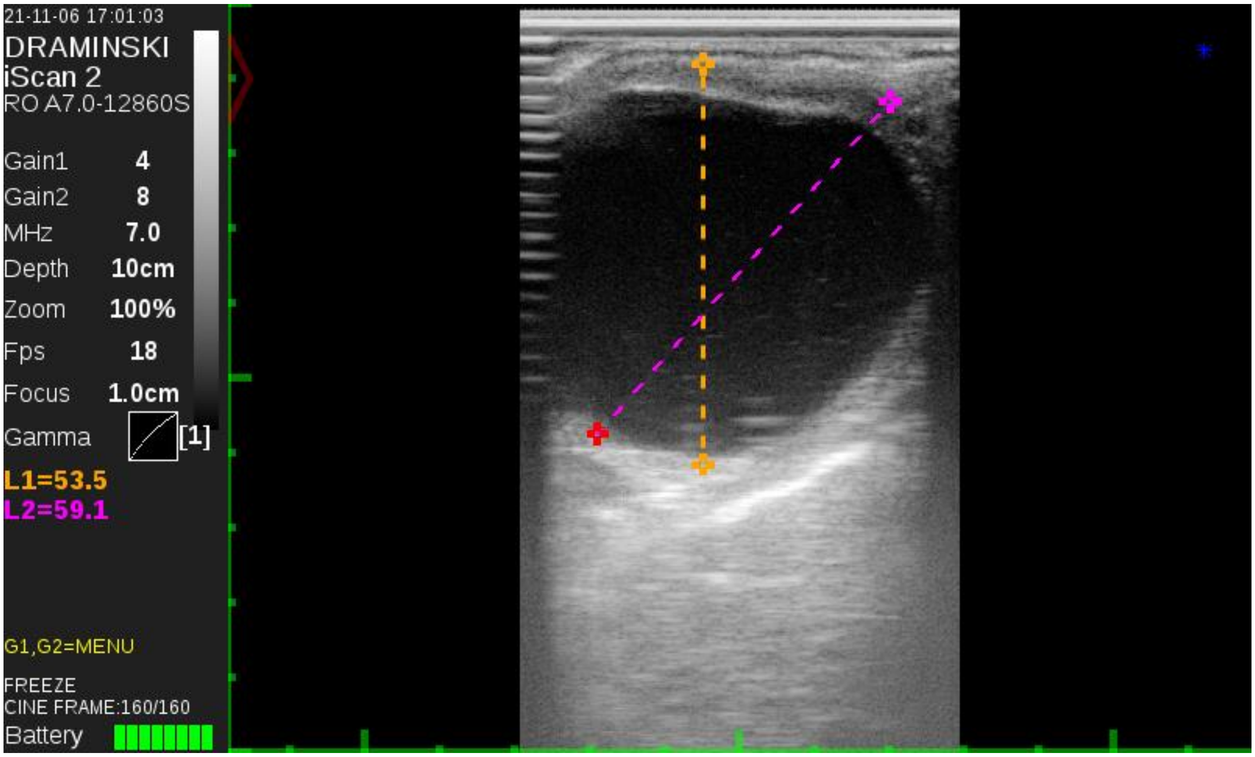

Pre-ovulatory follicle of a Percheron Mare (Recipient)

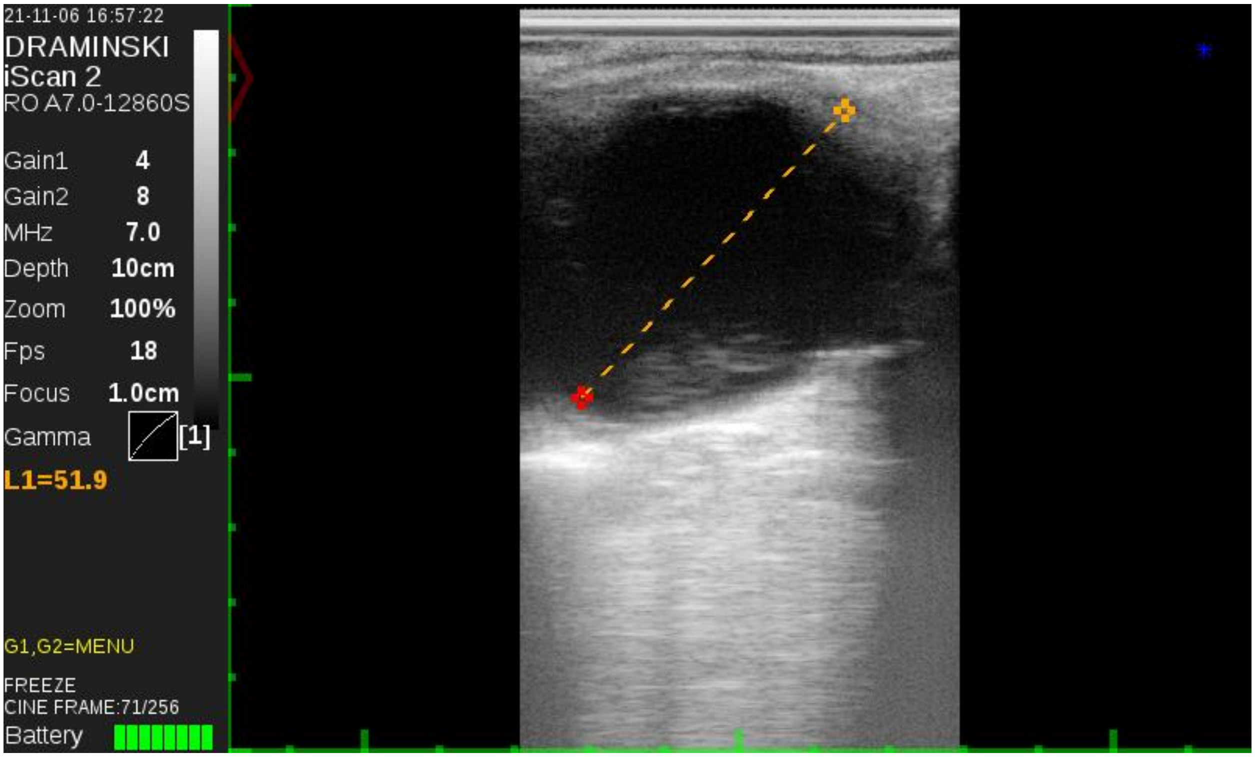

Pre-ovulatory follicle of a Percheron Mare (Recipient)

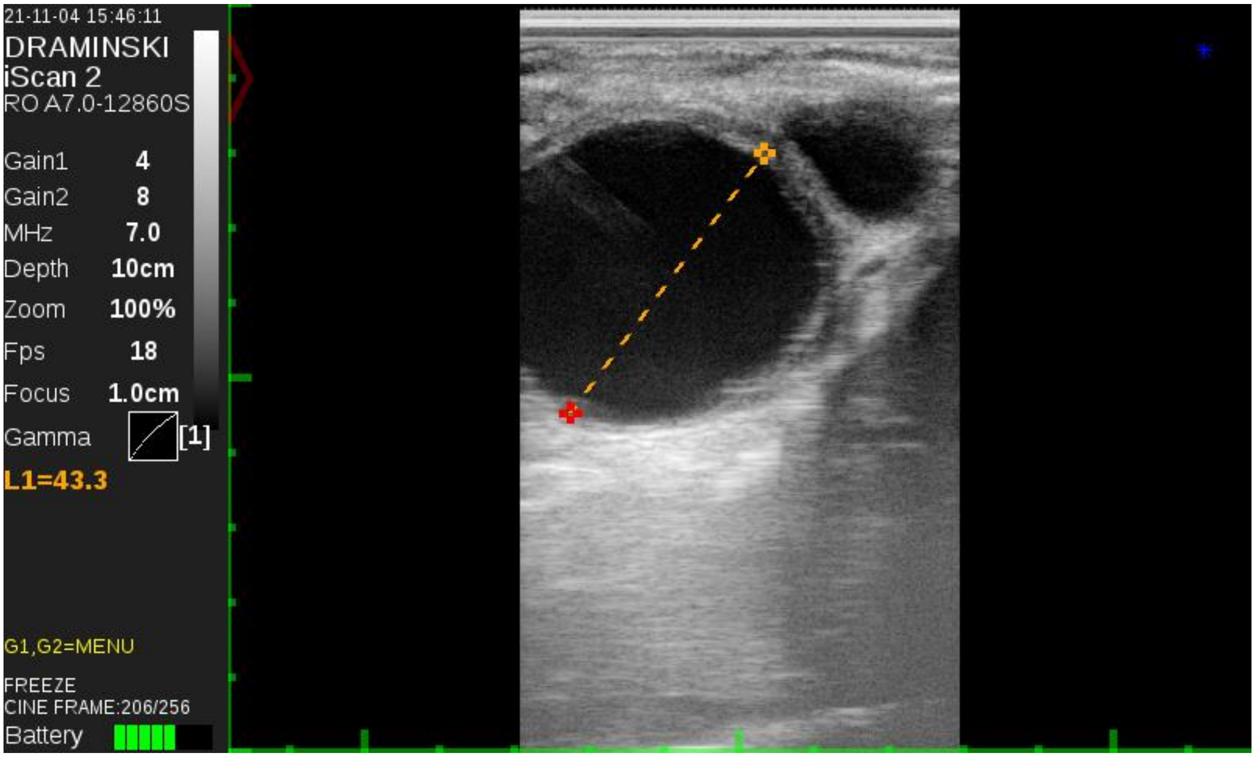

Pre-ovulatory follicle of an Argentino-Polo Mare (Donor)

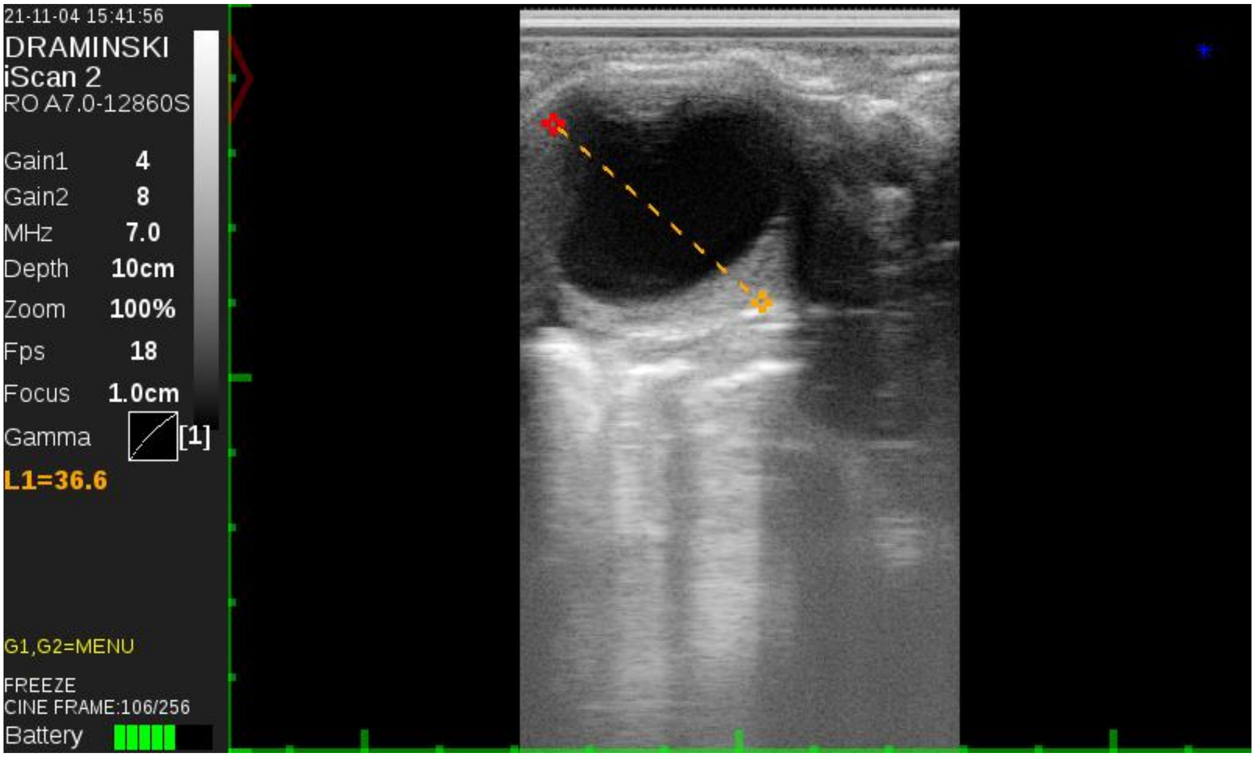

Pre-ovulatory follicle of an Anglo-Arab Mare (Donor)

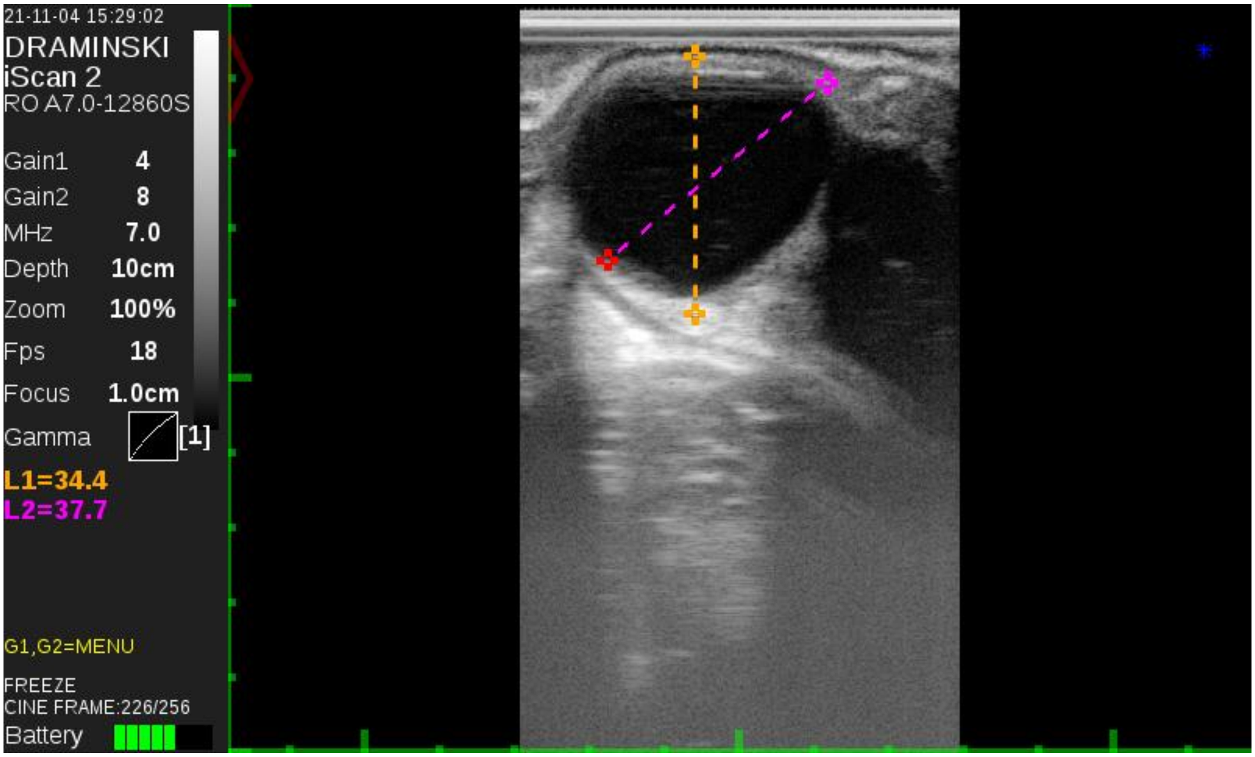

Pre-ovulatory follicle of an Anglo-Arab Mare (Donor)

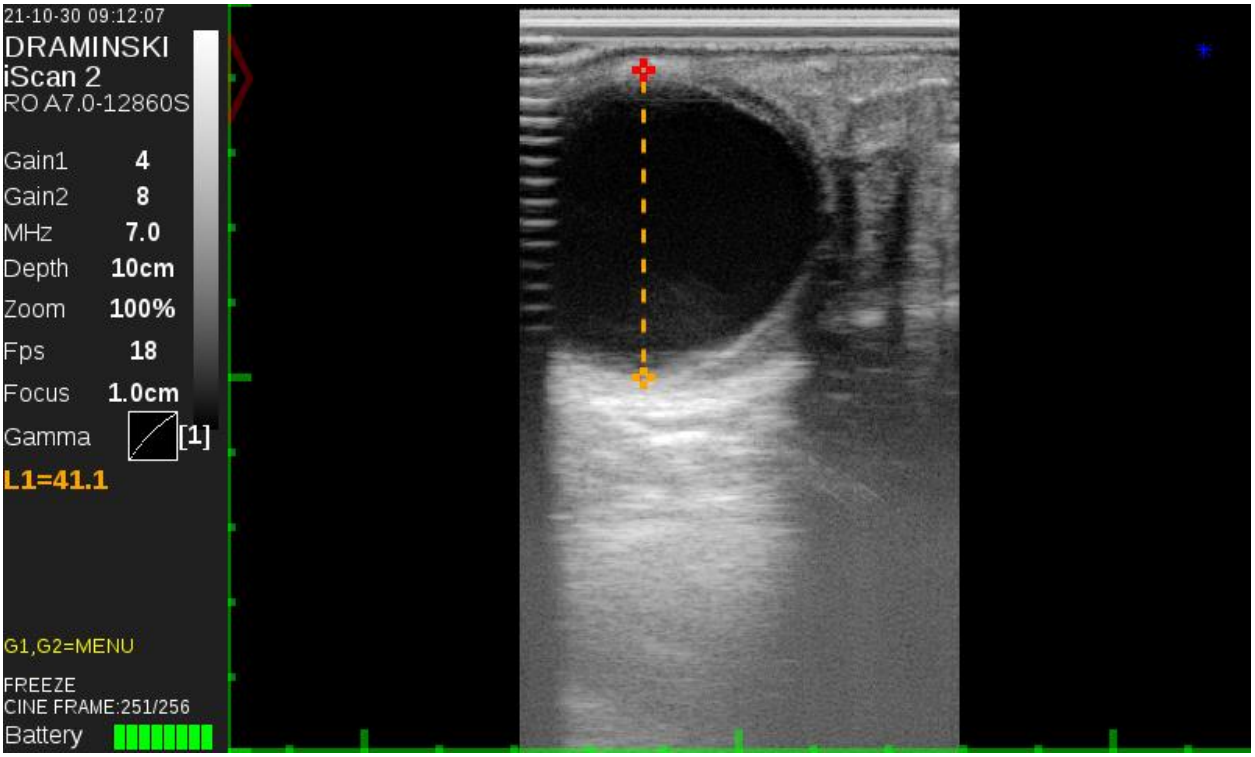

Pre-ovulatory follicle of a Light breed Mare (Recipient)

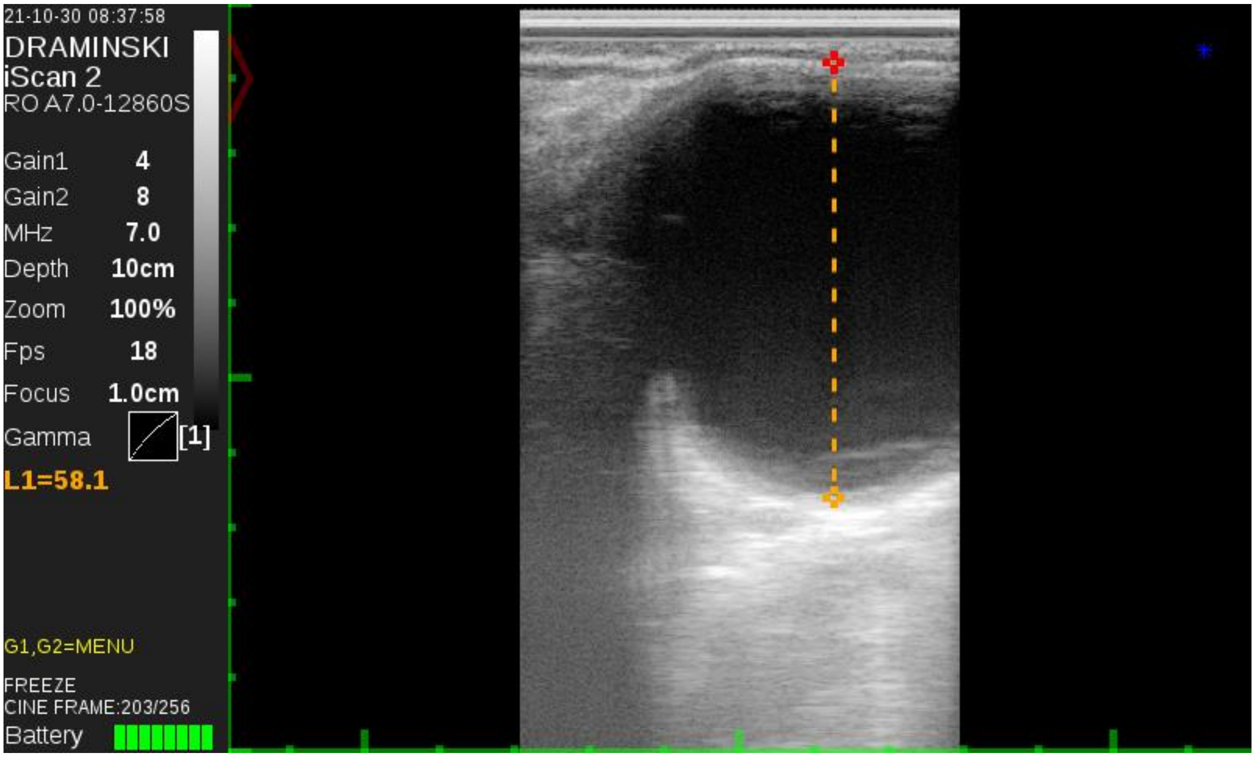

Pre-ovulatory follicle of a Percheron Mare (Recipient)

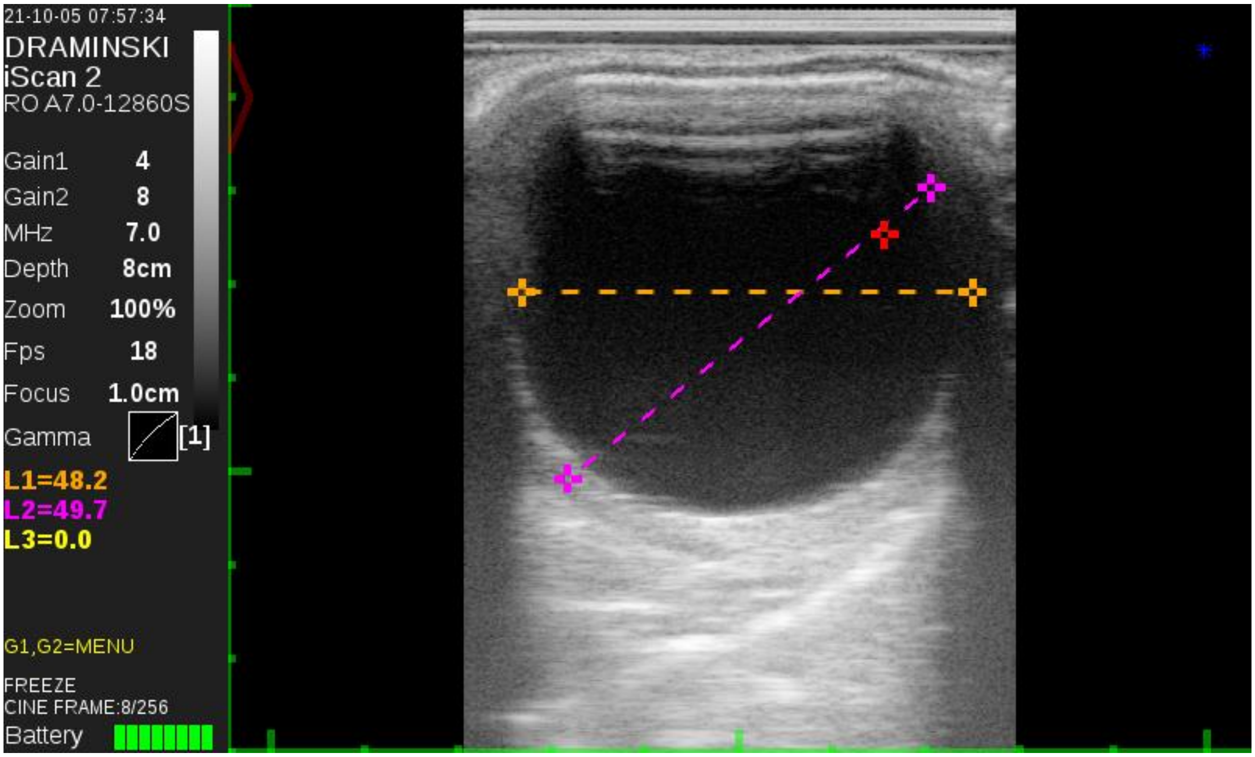

Pre-ovulatory follicle of a Percheron Mare (Recipient)

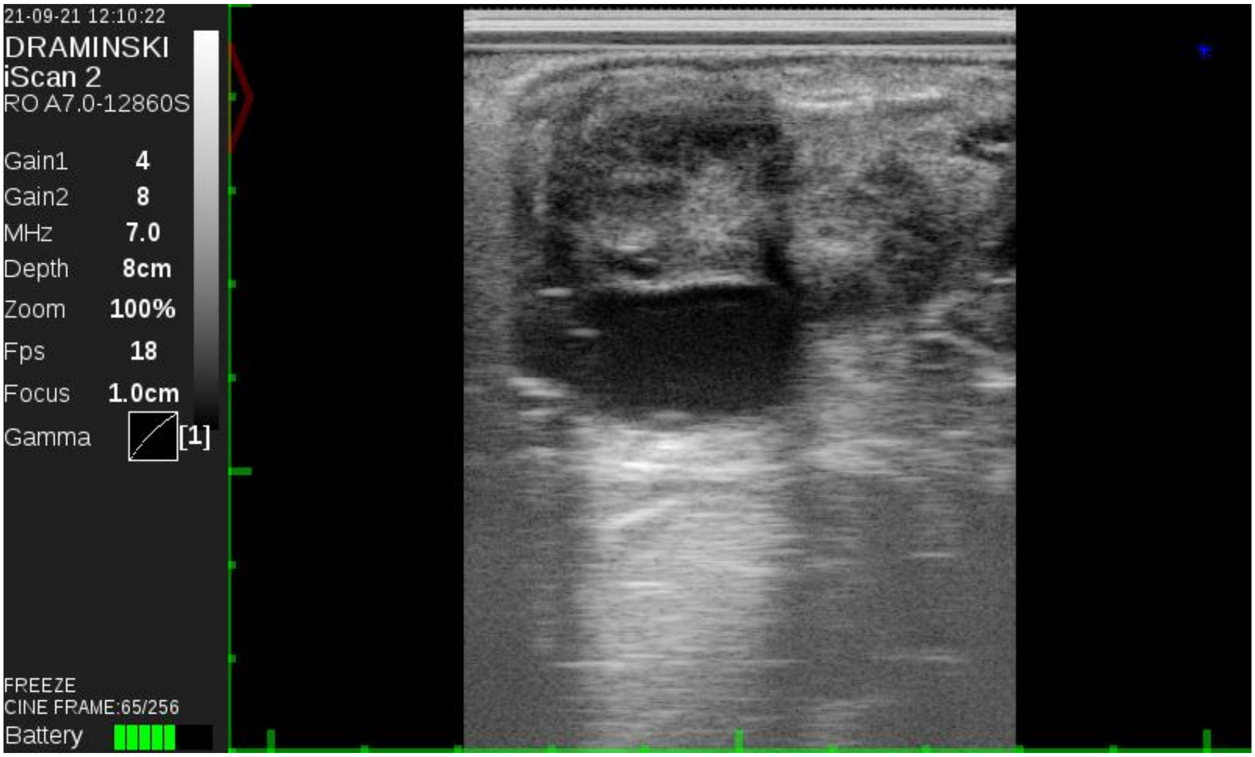

Corpus luteum a Percheron Mare (Recipient)

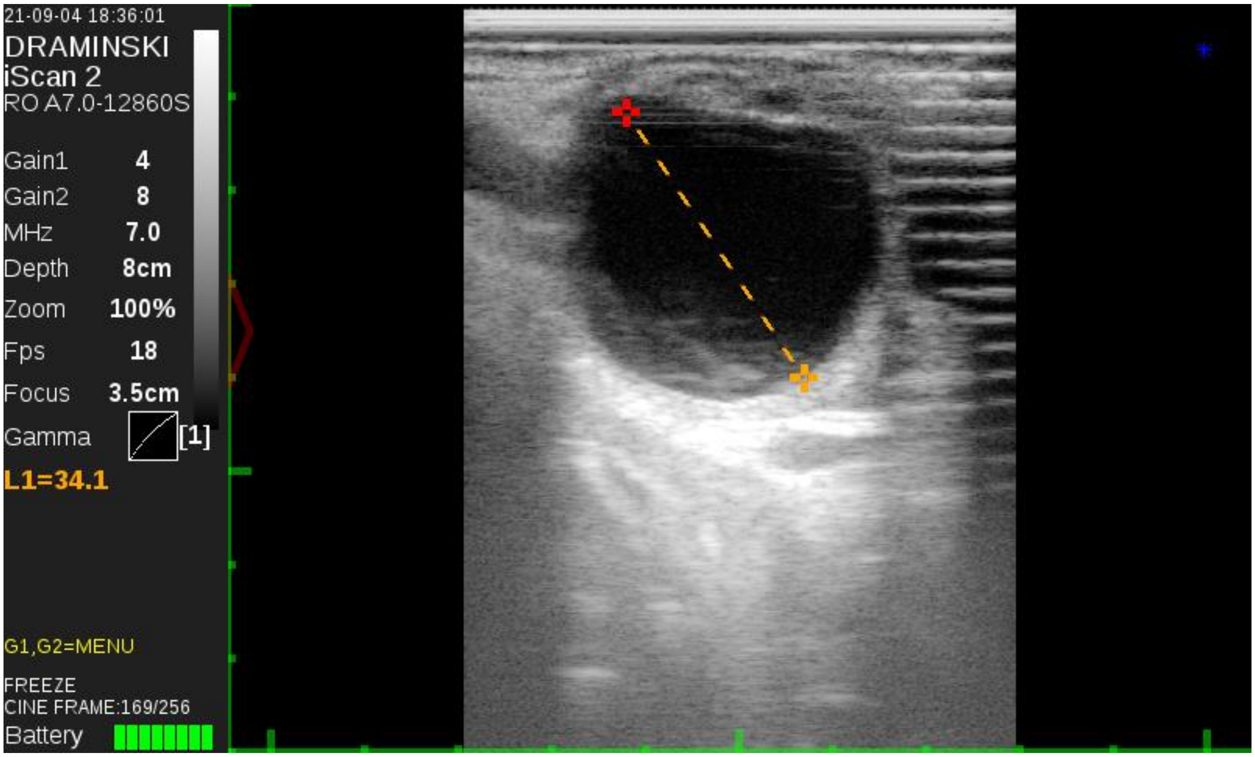

Large follicle of an Anglo-Arab Mare (Recipient)

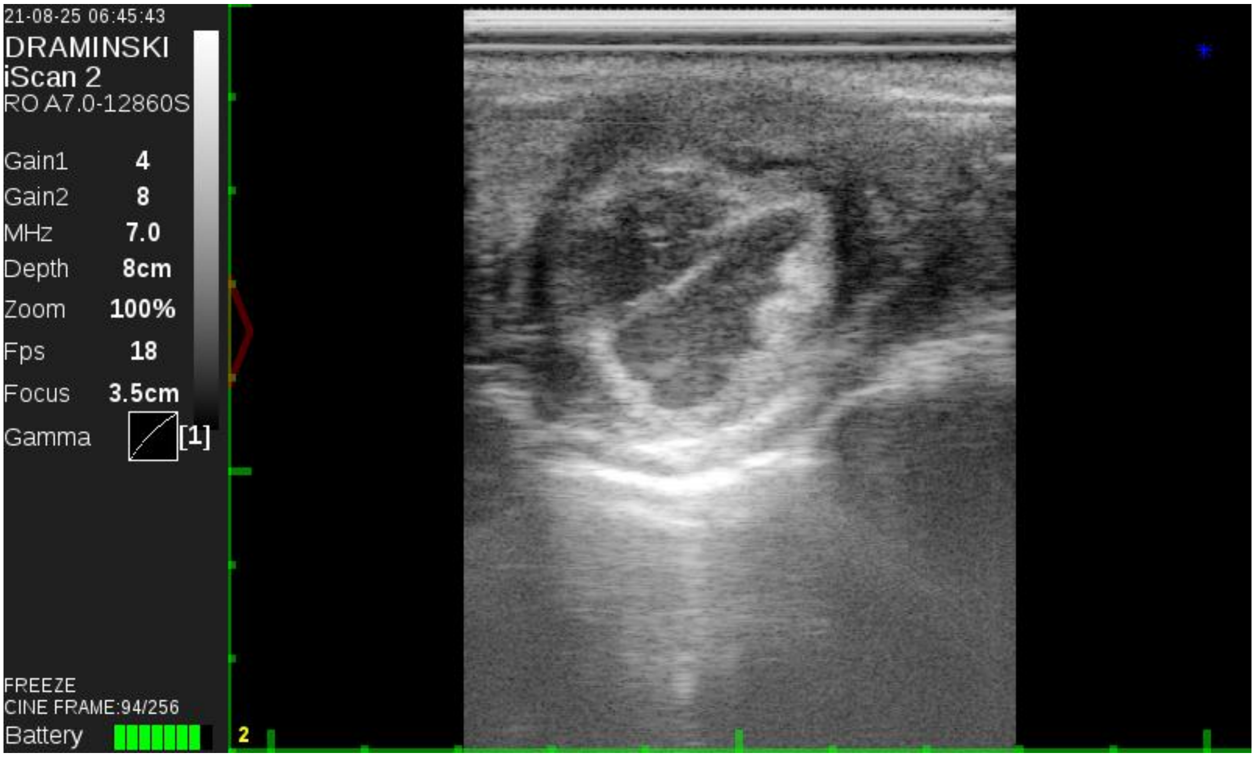

Corpora Hemorrhagica (C.H) of a Percheron Mare (Recipient)

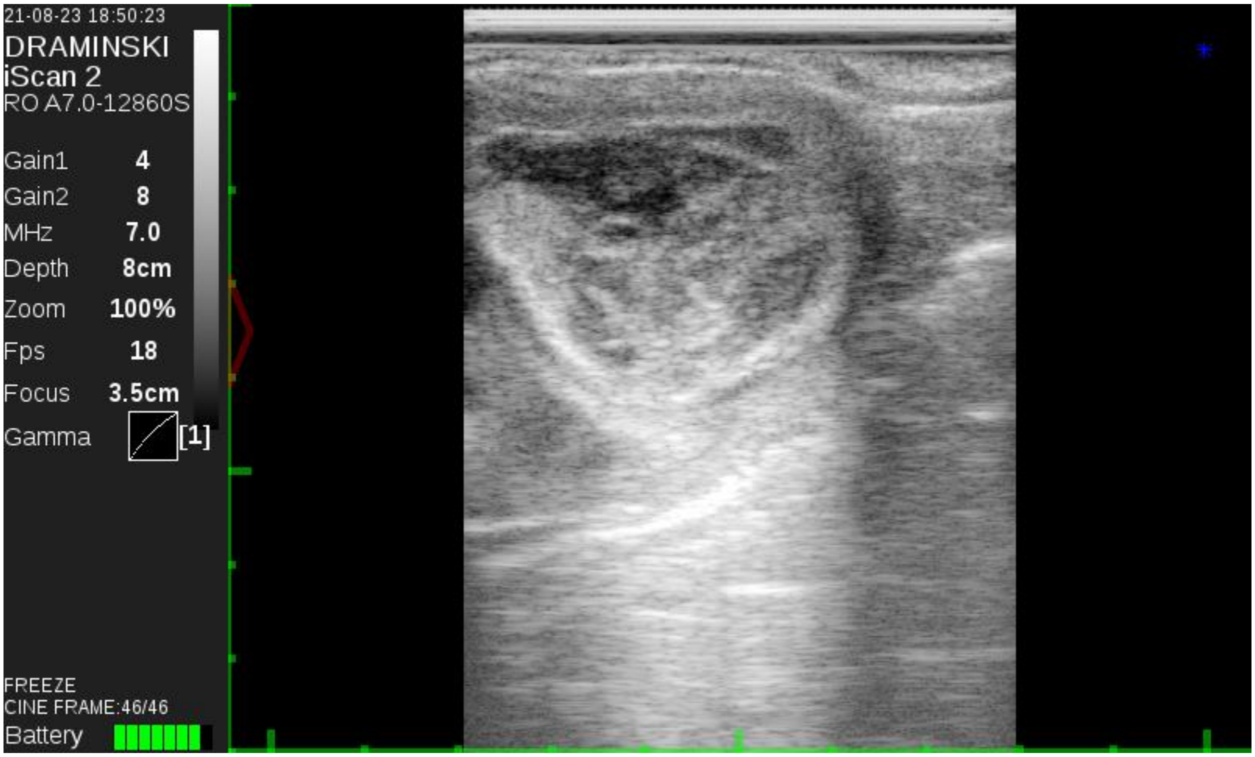

Corpora Hemorrhagica (C.H) of a Percheron Mare (Recipient)

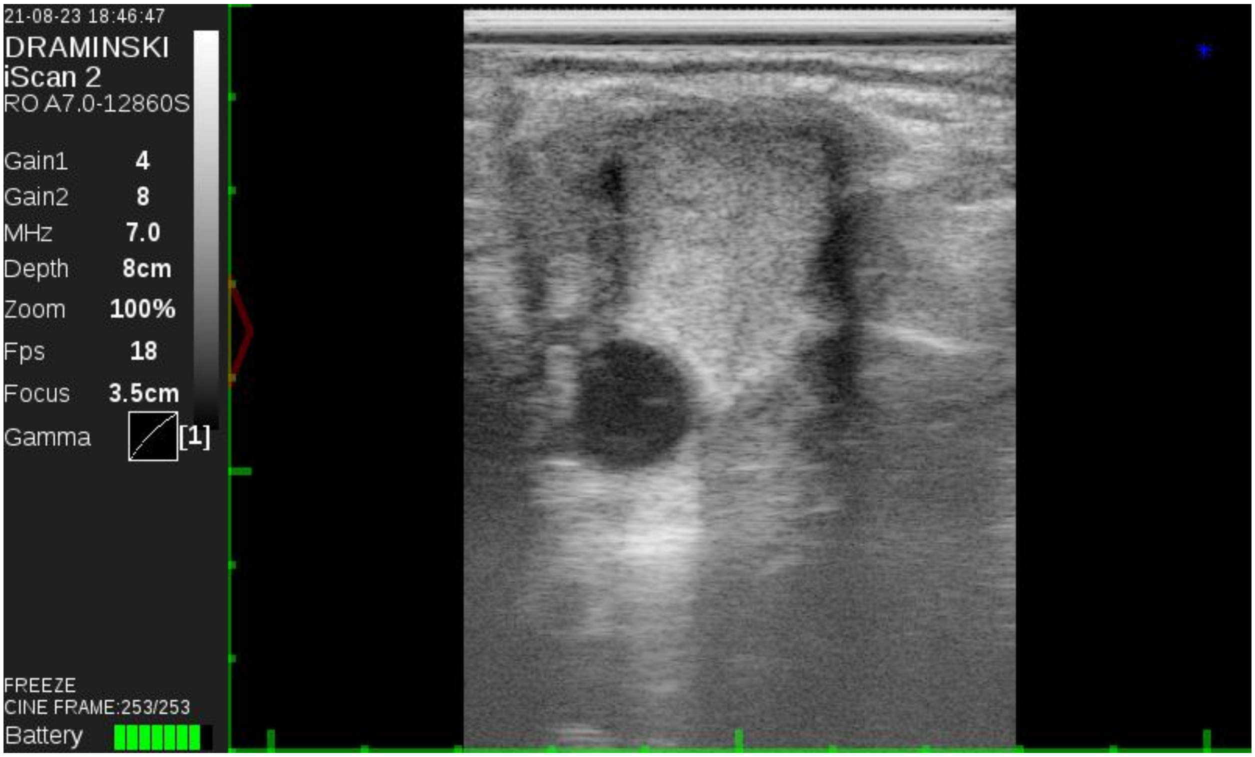

Corpora Luteum (CL) of an Anglo-Arab mare (Donor)

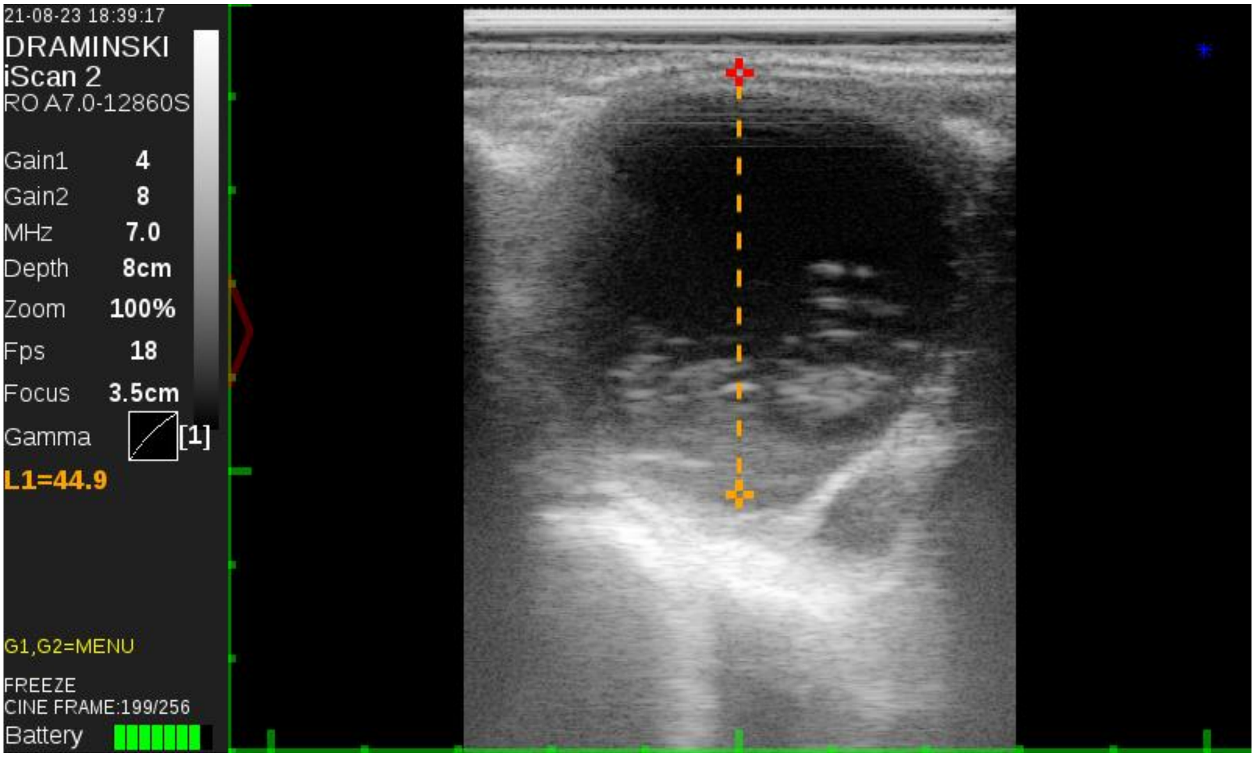

Pre-ovulatory follicle of a Percheron Mare (Recipient)

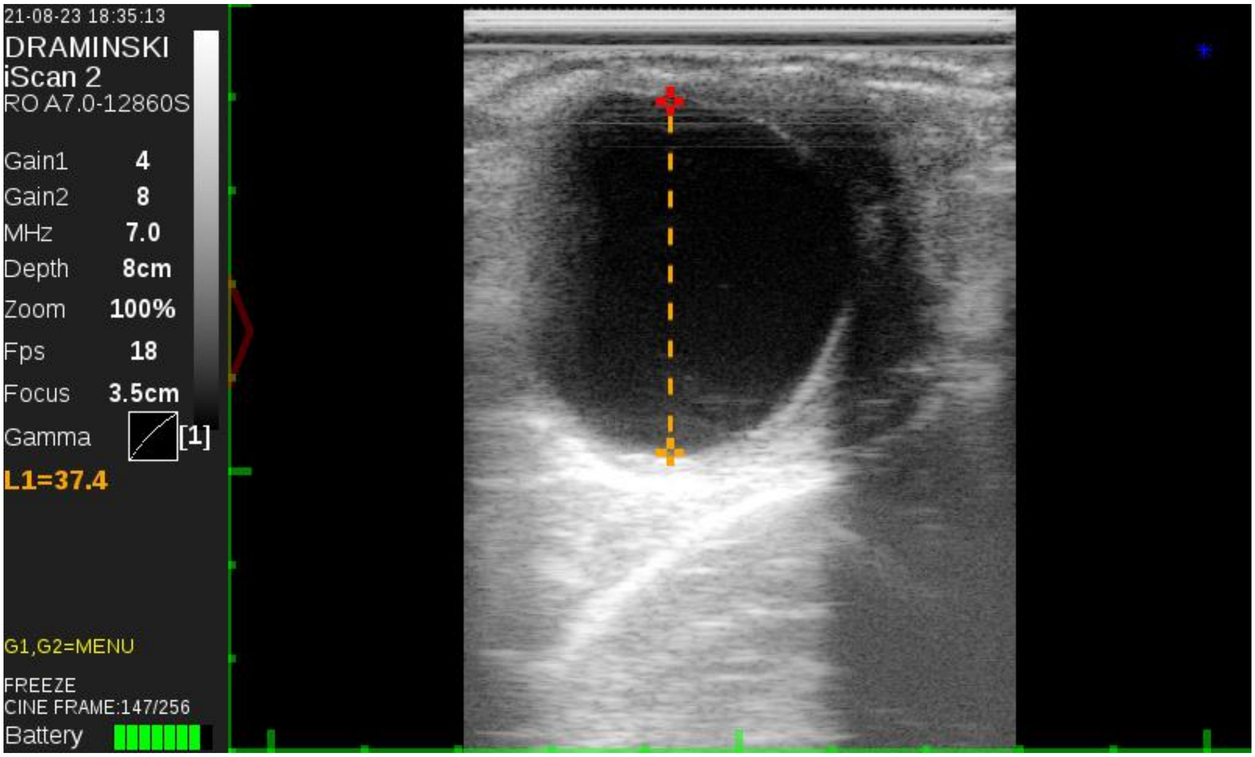

Pre-ovulatory of an Argentino-Polo mare (Donor)

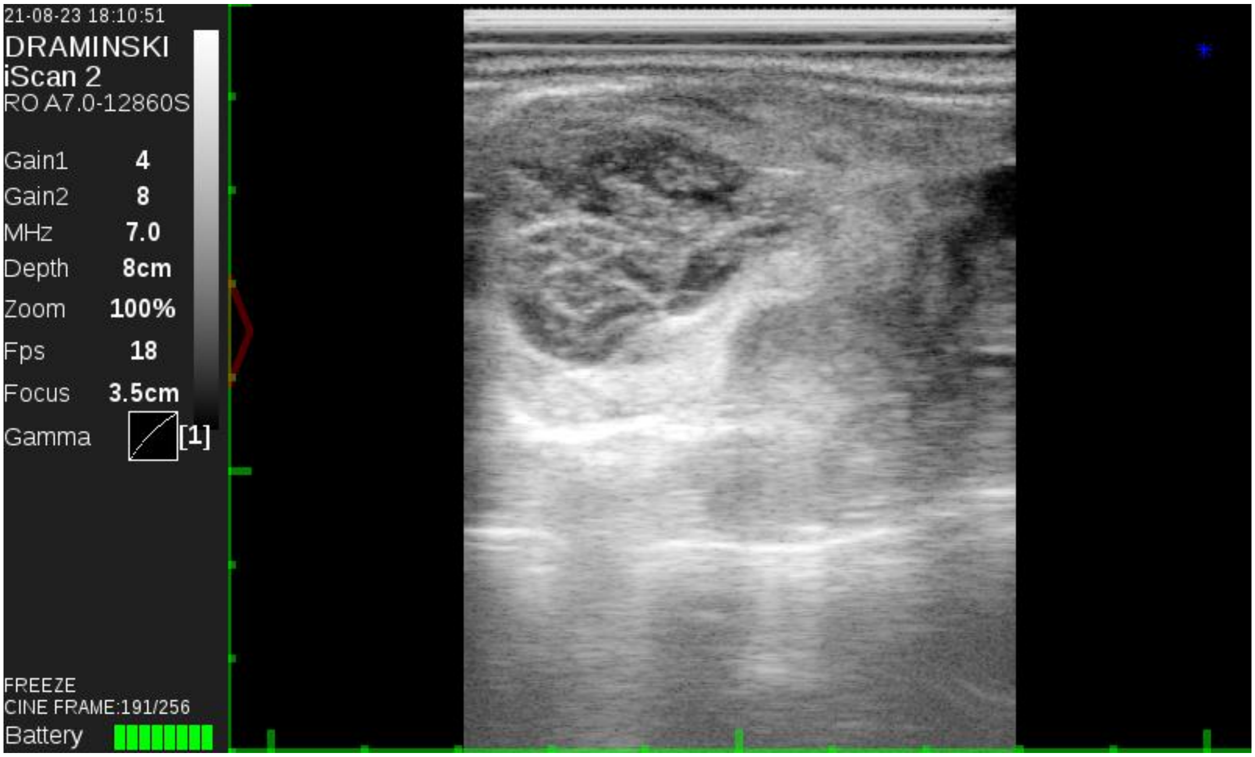

Corpora Hemorrhagica (C.H) of an Argentino-Polo Mare (Donor)

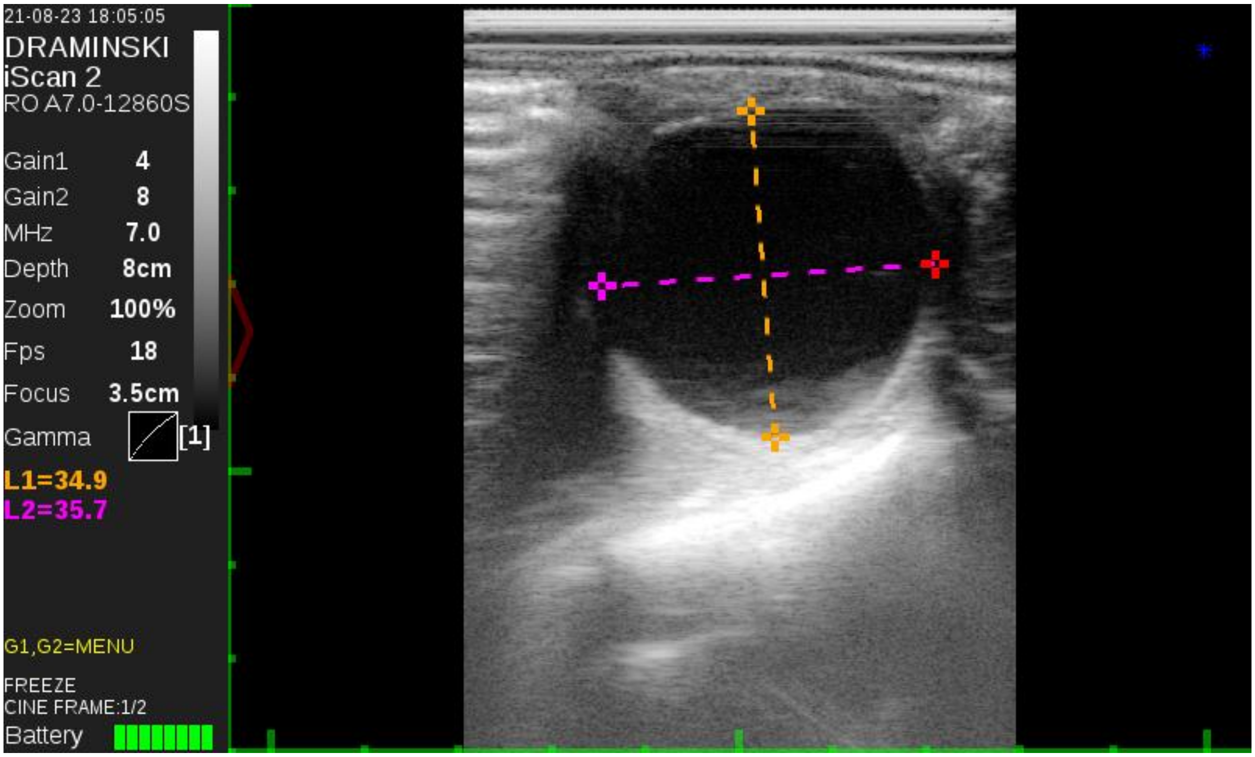

<u>**Pre-ovulatory follicle of an Anglo-arab Mare (Donor)**</u>

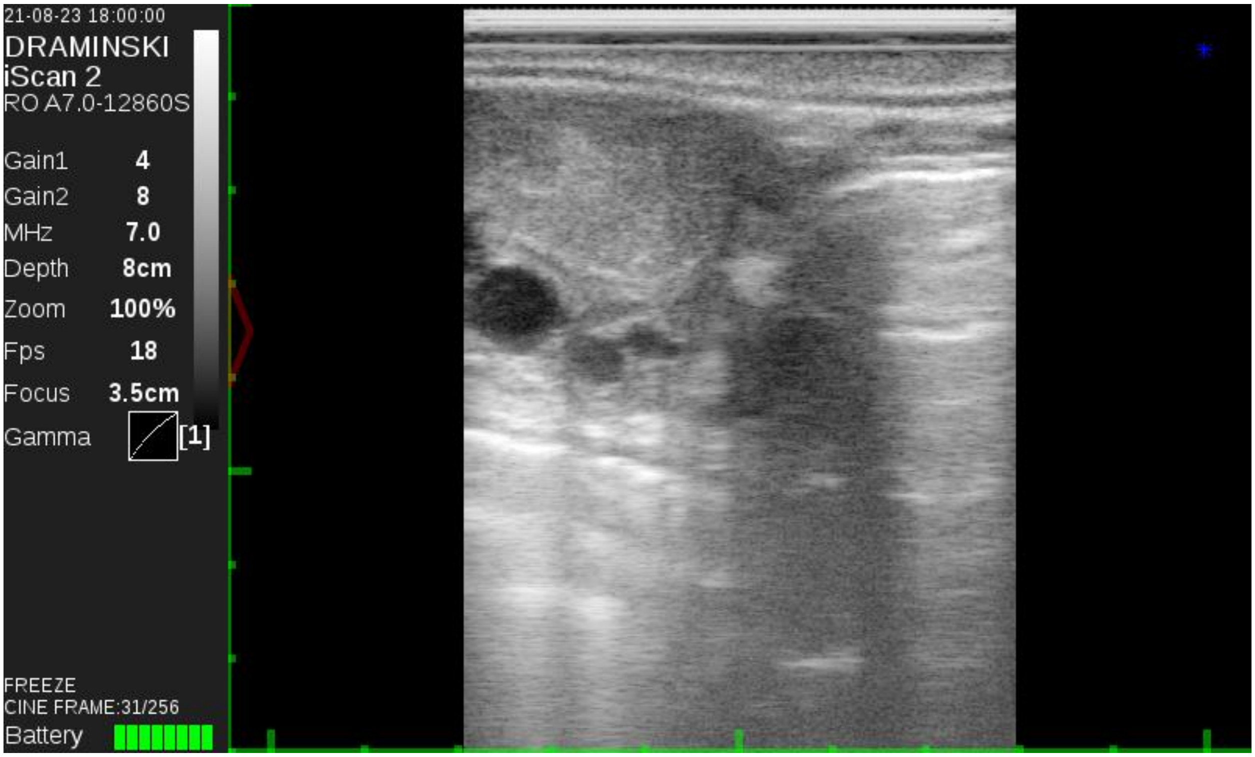

<u>**Corpus Luteum (CL) of an Anglo-Arab Mare (Donor)**</u>

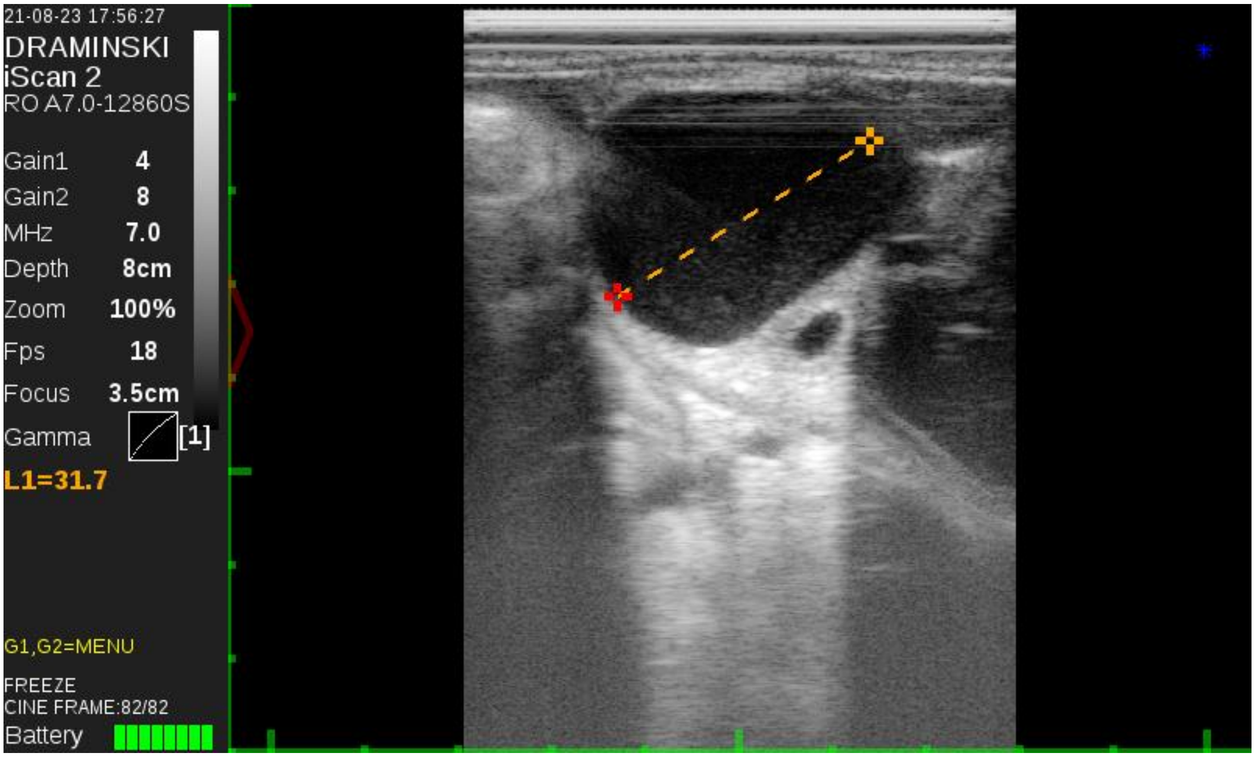

<u>**Growing follicle of a Light breed Mare (Recipient)**</u>

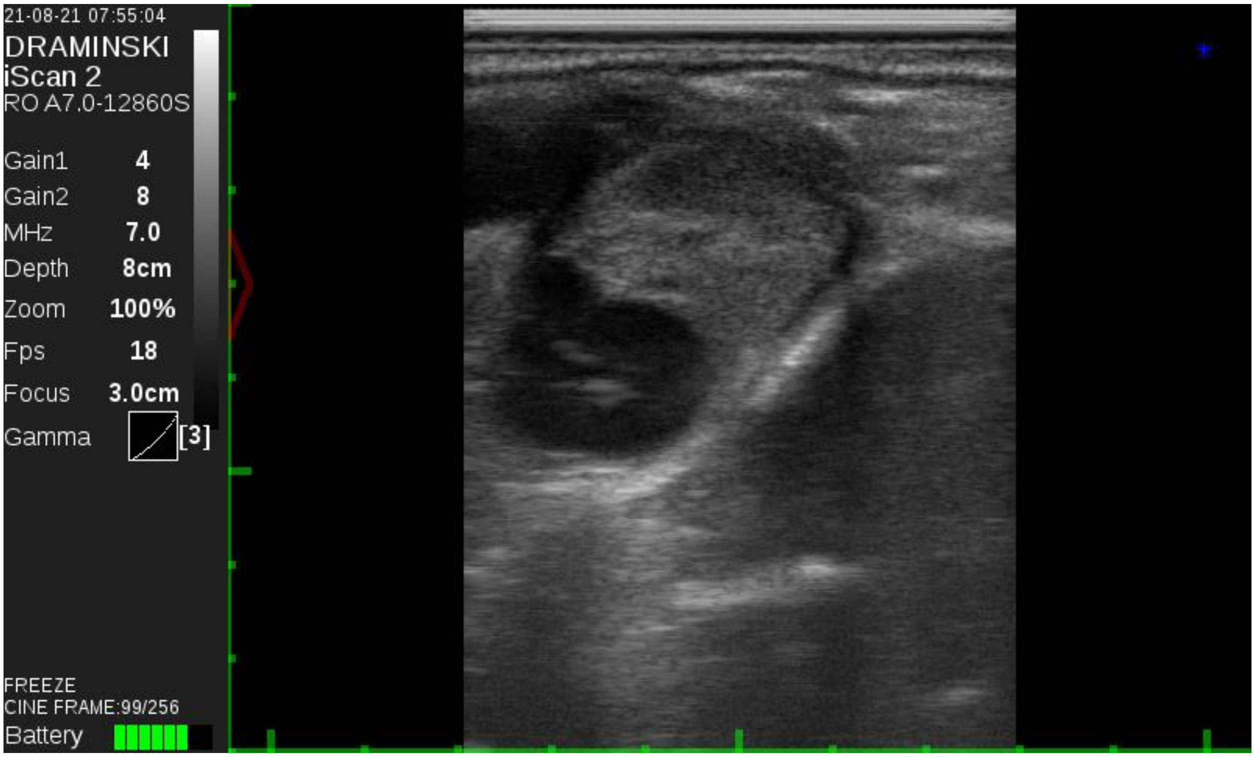

<u>**Corpus luteum (CL) of a Light breed Mare (Recipient)**</u>
