# Supplementary material for: Factors affecting the efficiency of equine embryo transfer (EET) in polo mares under subtropical conditions of Pakistan
Source: PLoS One. 2024 Feb 12;19(2):e0298066. doi: 10.1371/journal.pone.0298066 (PMC10861068; doi:10.1371/journal.pone.0298066)
Supplement: S4 Fig — (PDF) [file pone.0298066.s006.pdf]

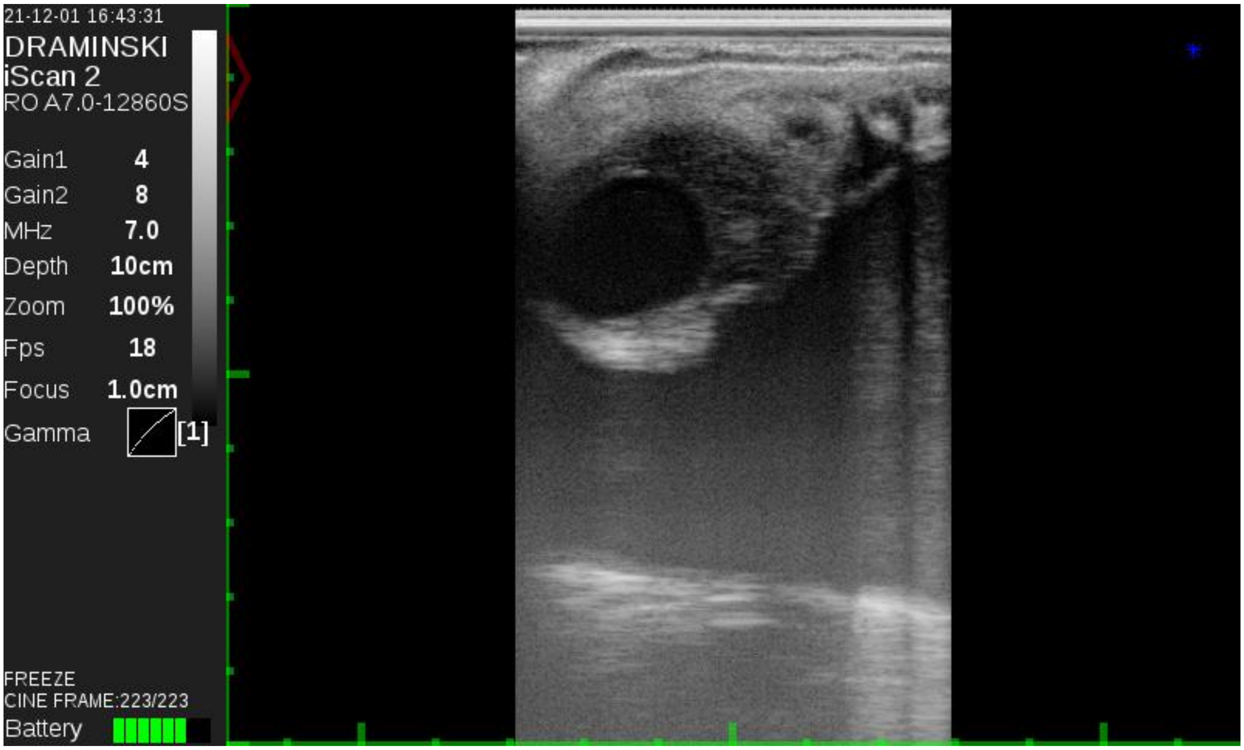

14 Days Pregnancy

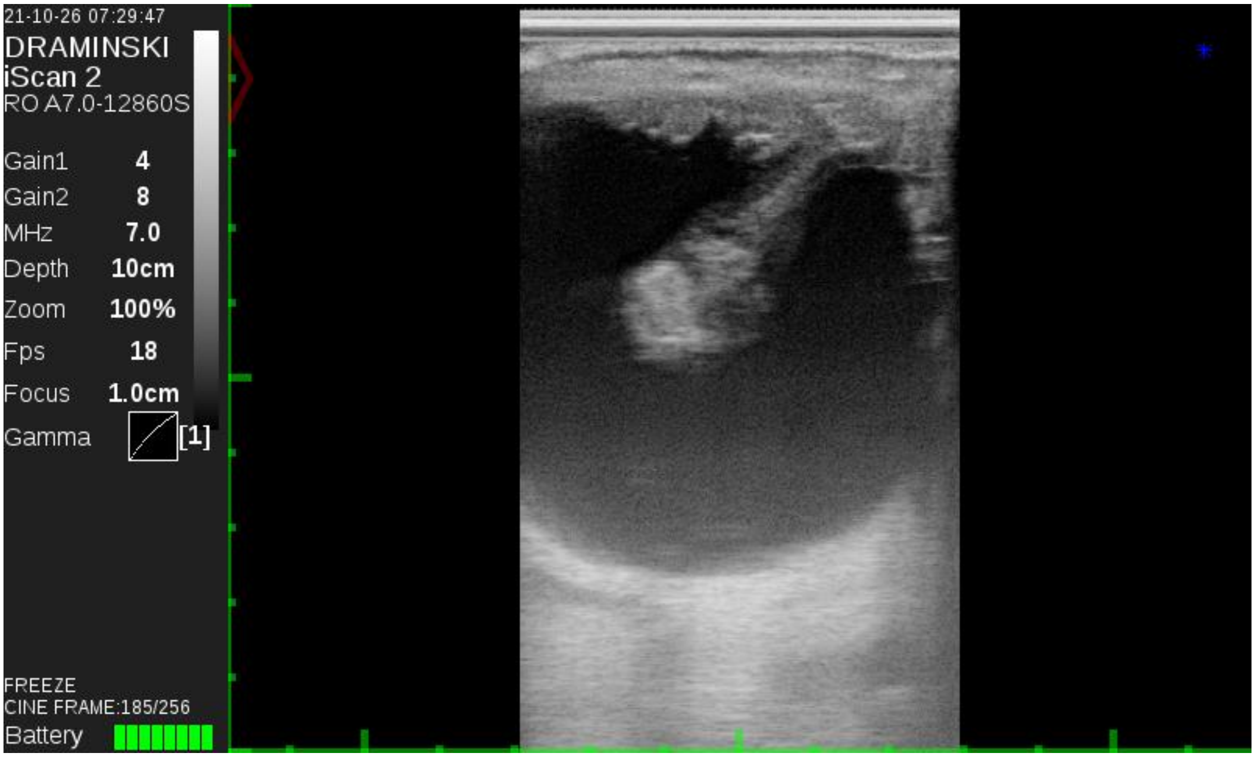

35 Days Pregnancy

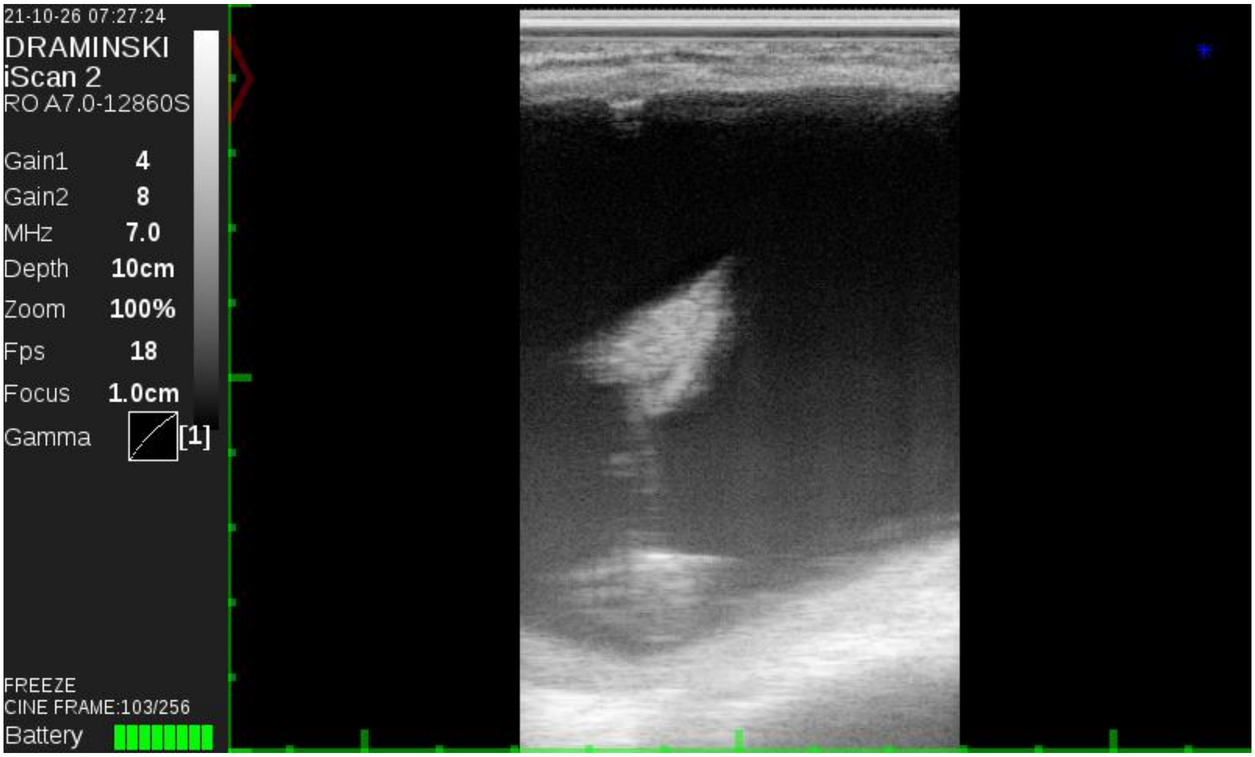

40 Days Pregnancy

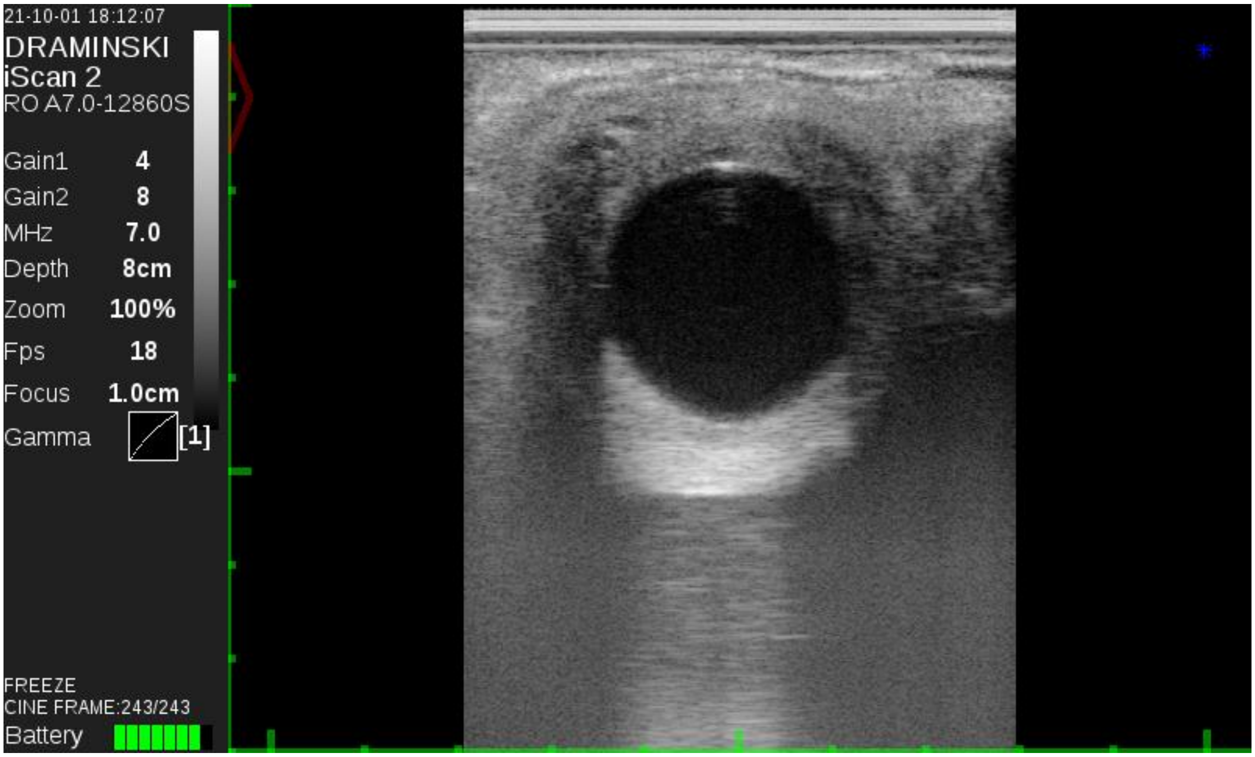

14 Days Pregnancy

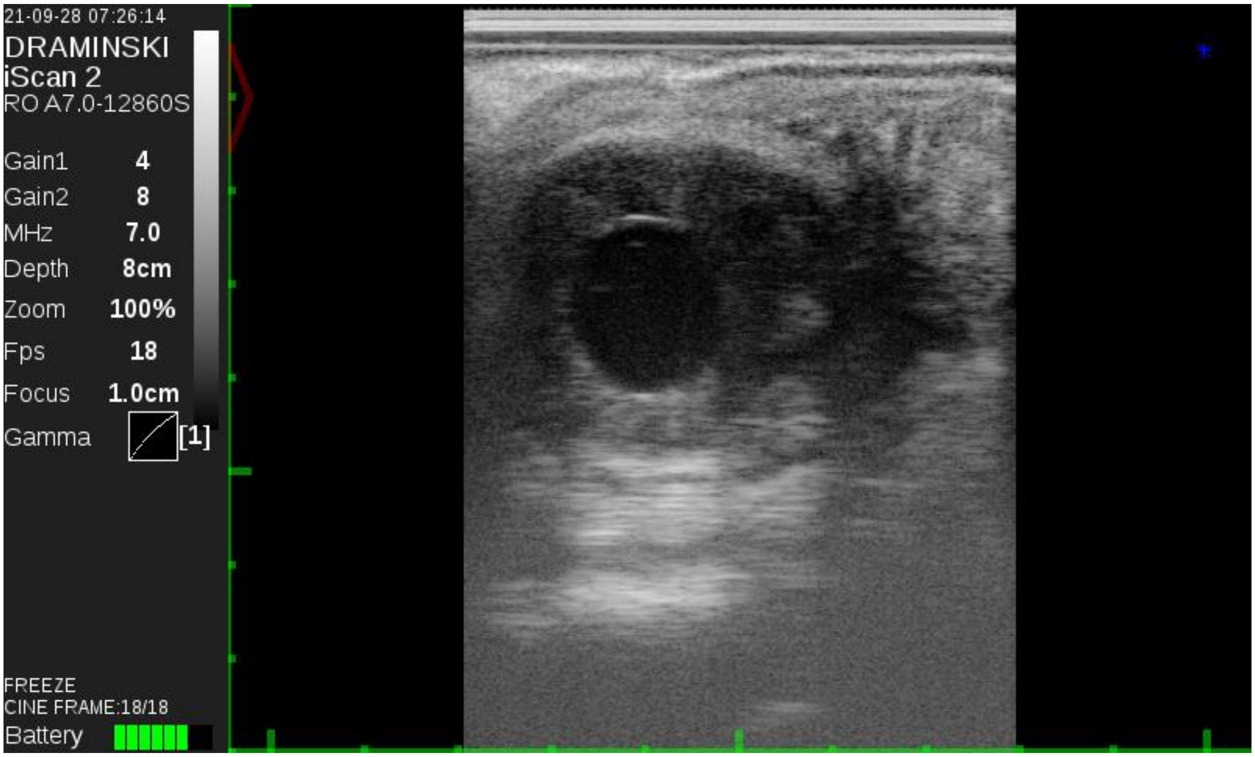

14 Days Pregnancy

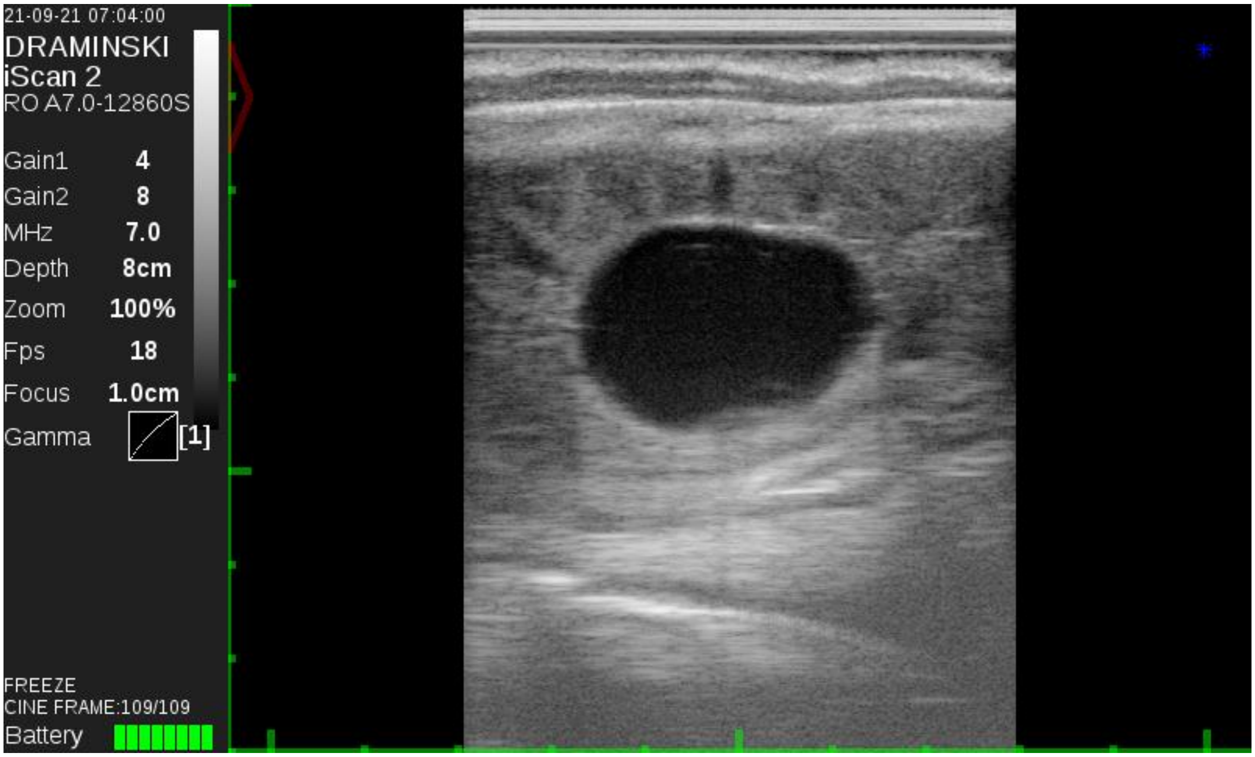

20 Days Pregnancy

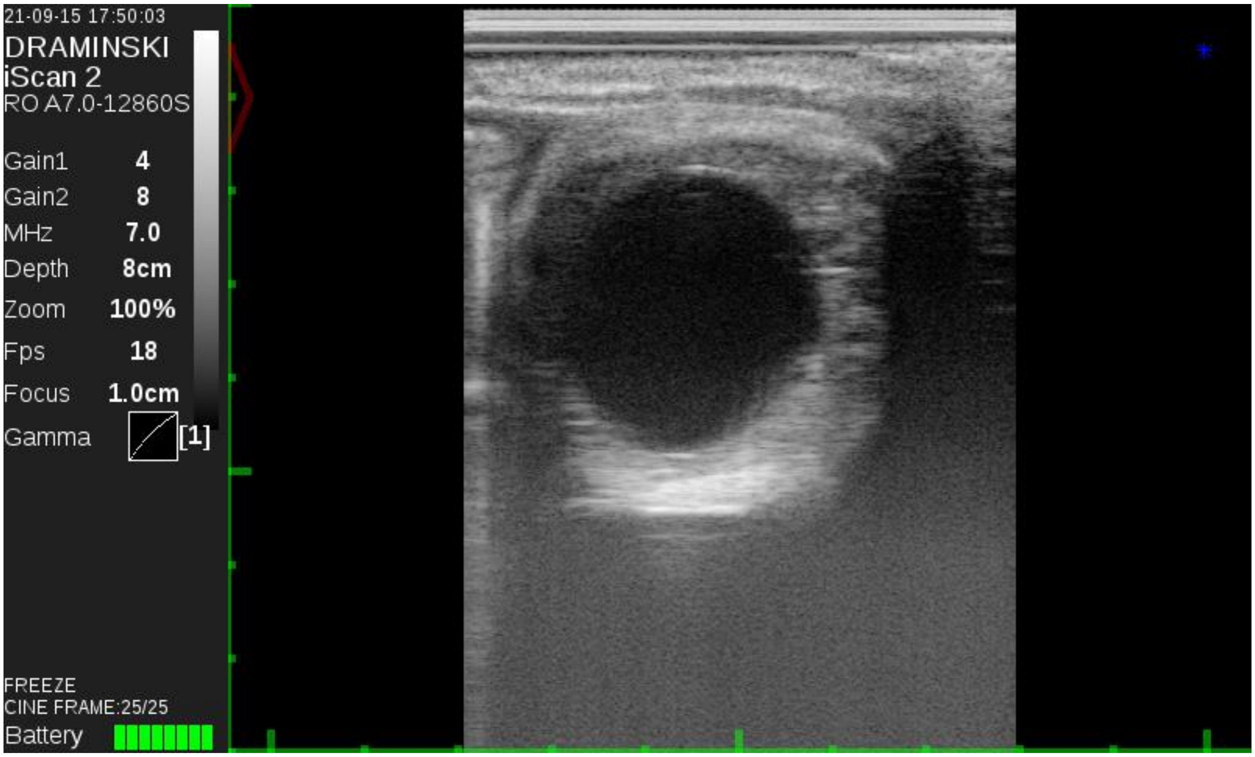

14 Days Pregnancy

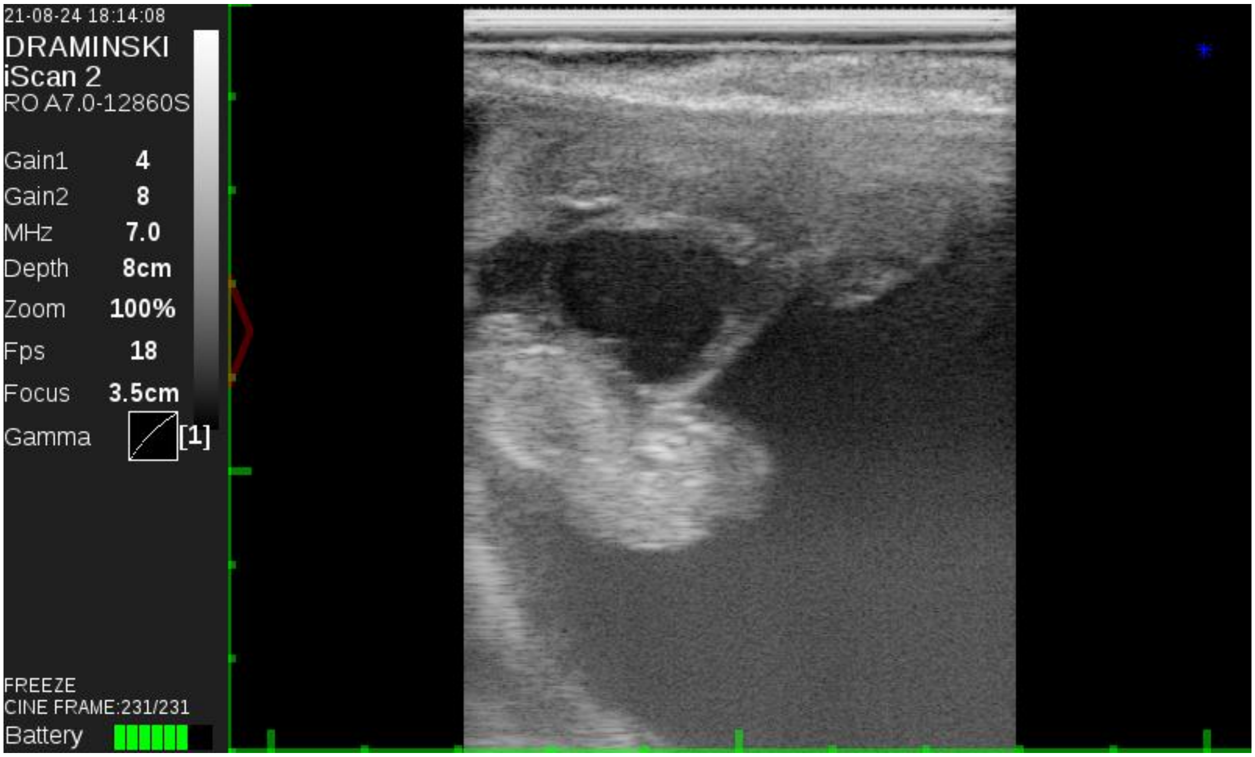

50 Days Pregnancy

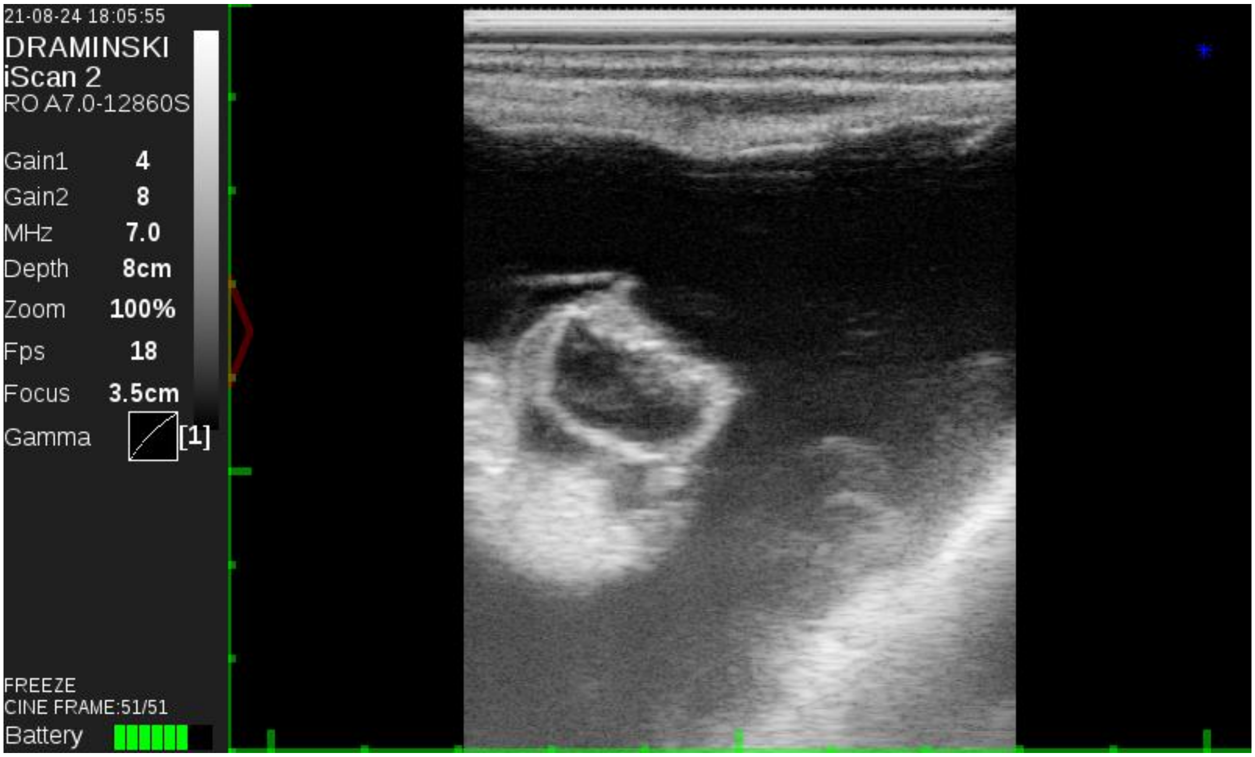

50 Days Pregnancy

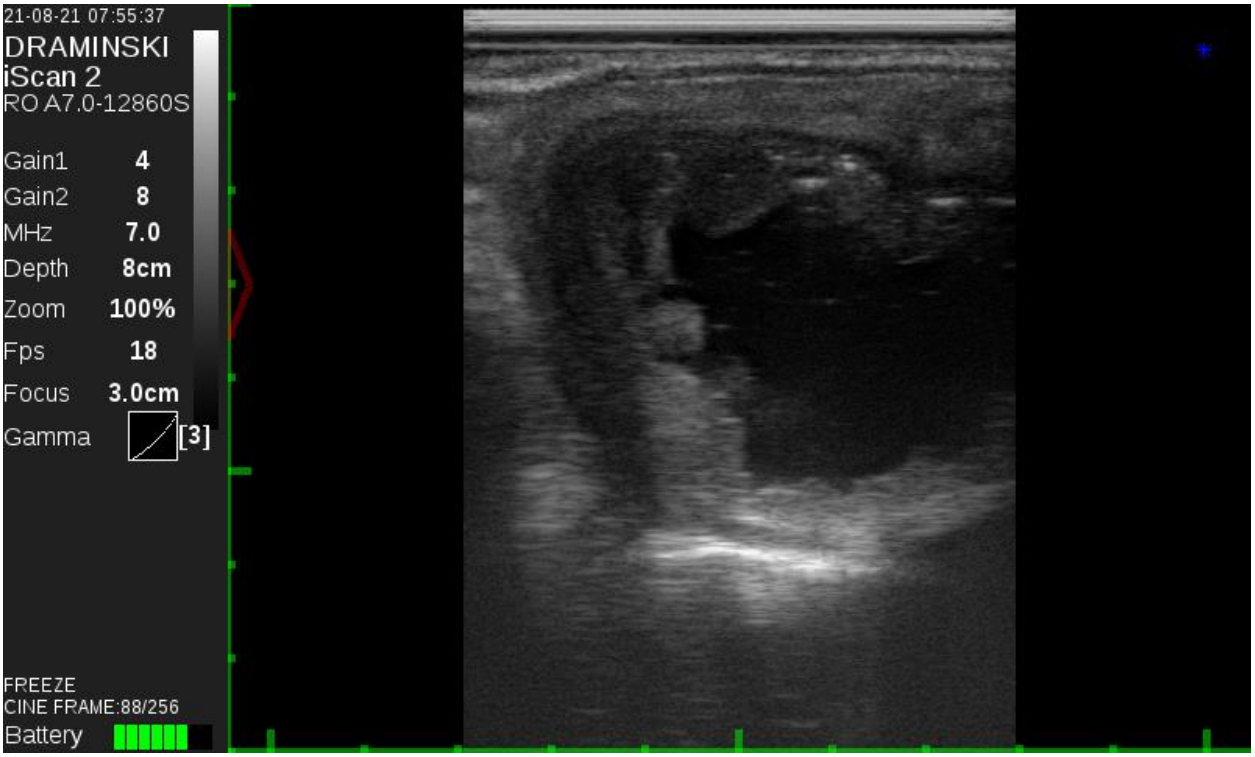

22 Days Pregnancy

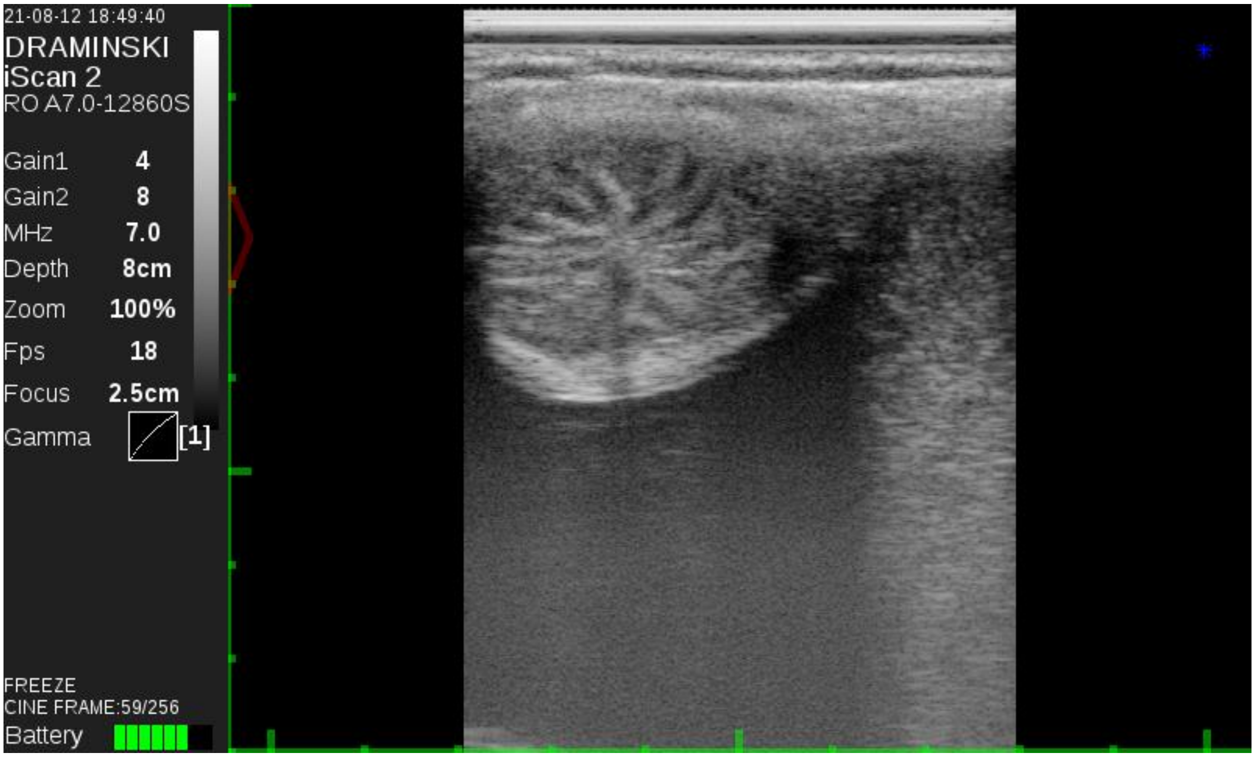

Sligh uterine edema (Edema Score-1)

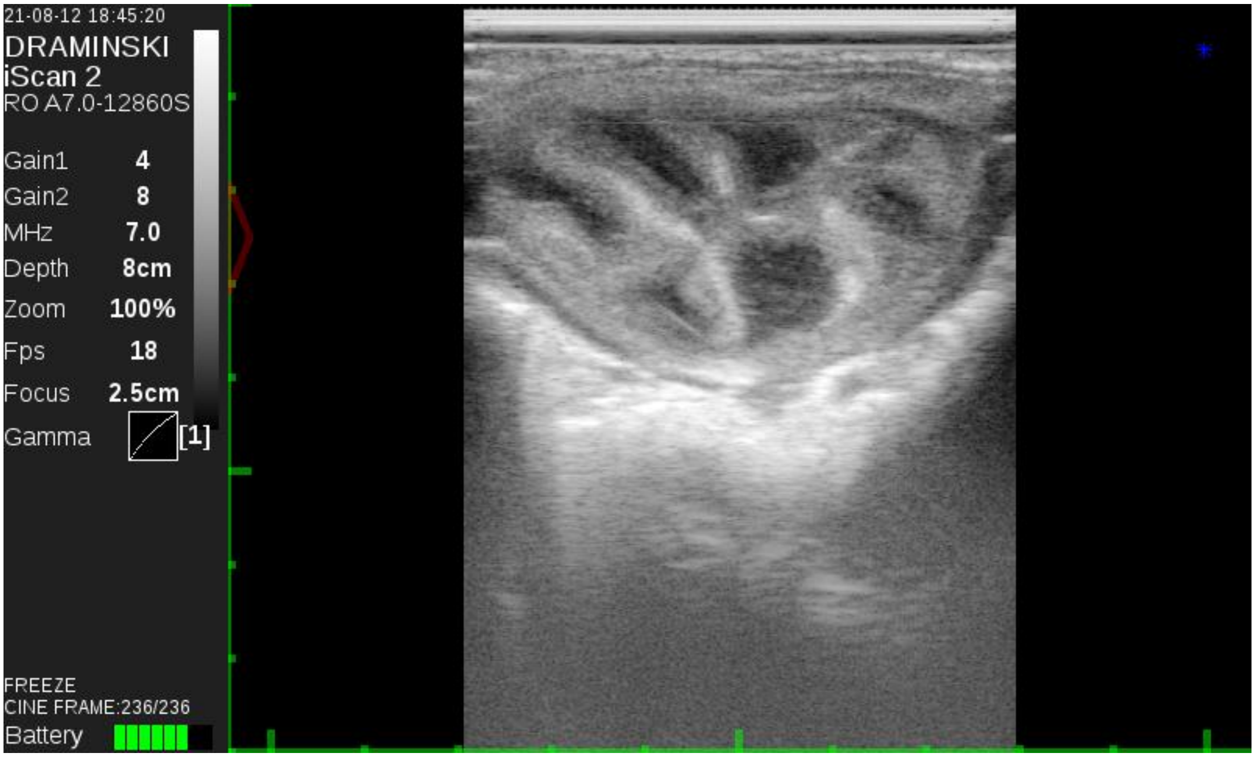

Moderate uterine edema (Edema Score-2)

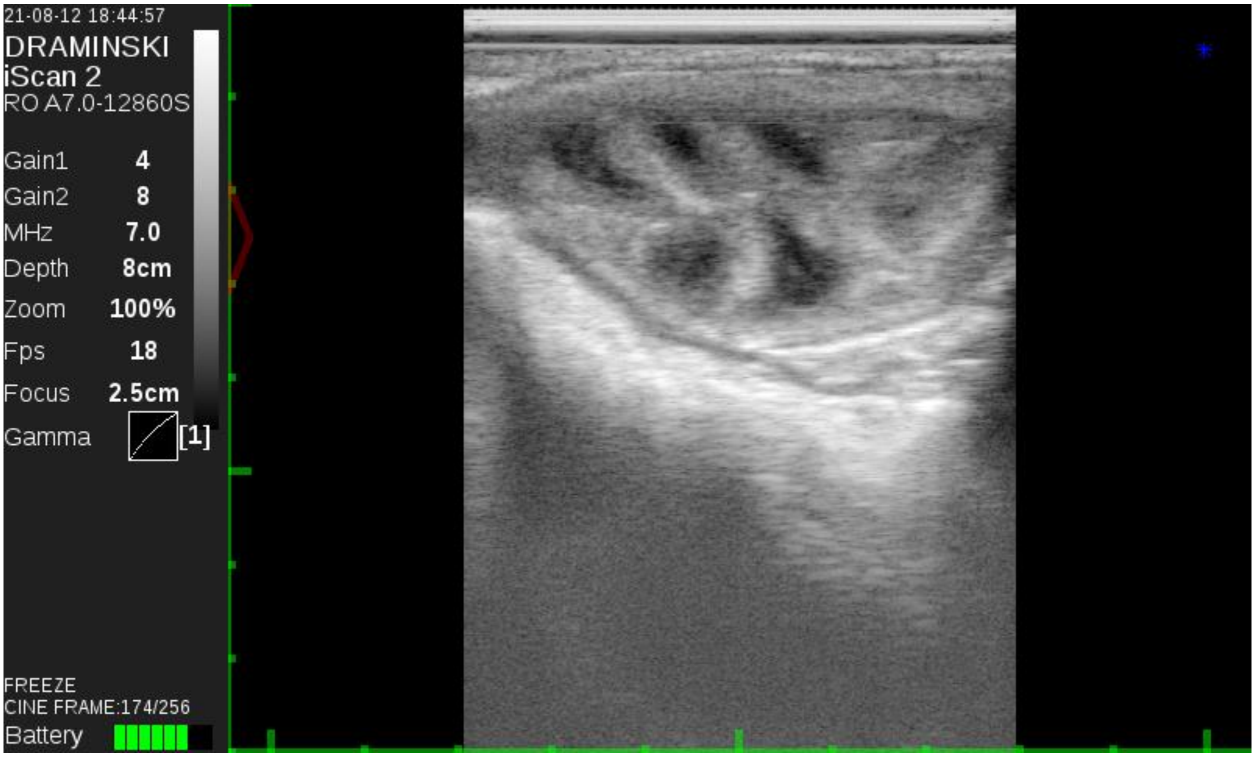

Moderate uterine edema (Edema Score-2)
