# Supplementary material for: Factors affecting the efficiency of equine embryo transfer (EET) in polo mares under subtropical conditions of Pakistan
Source: PLoS One. 2024 Feb 12;19(2):e0298066. doi: 10.1371/journal.pone.0298066 (PMC10861068; doi:10.1371/journal.pone.0298066)
Supplement: S5 Fig — (PDF) [file pone.0298066.s007.pdf]

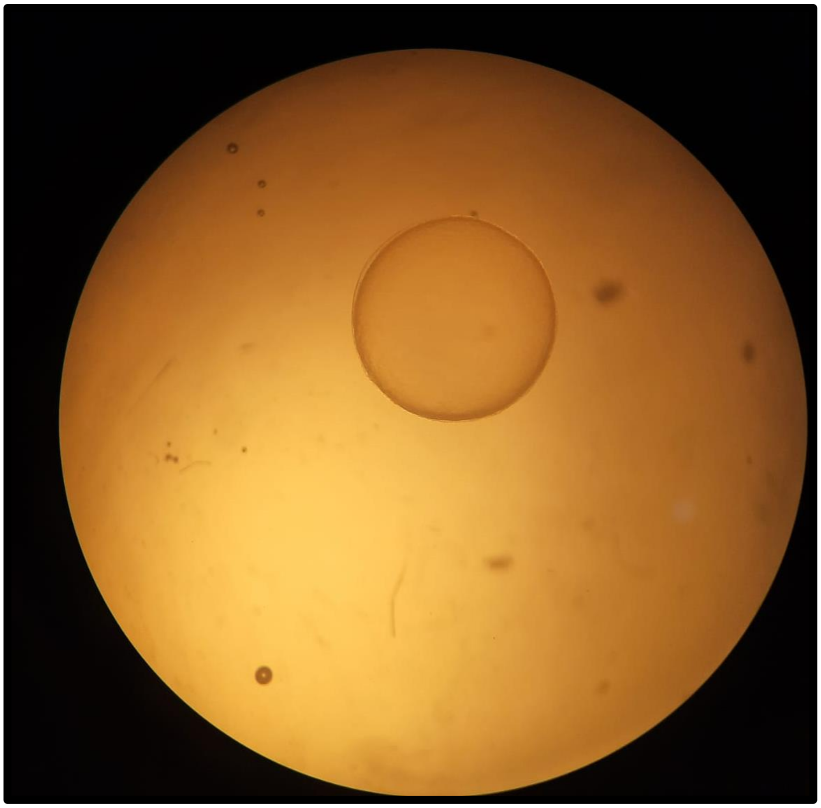

Day – 8 Mare Embryo (Expanded Blastocyst)

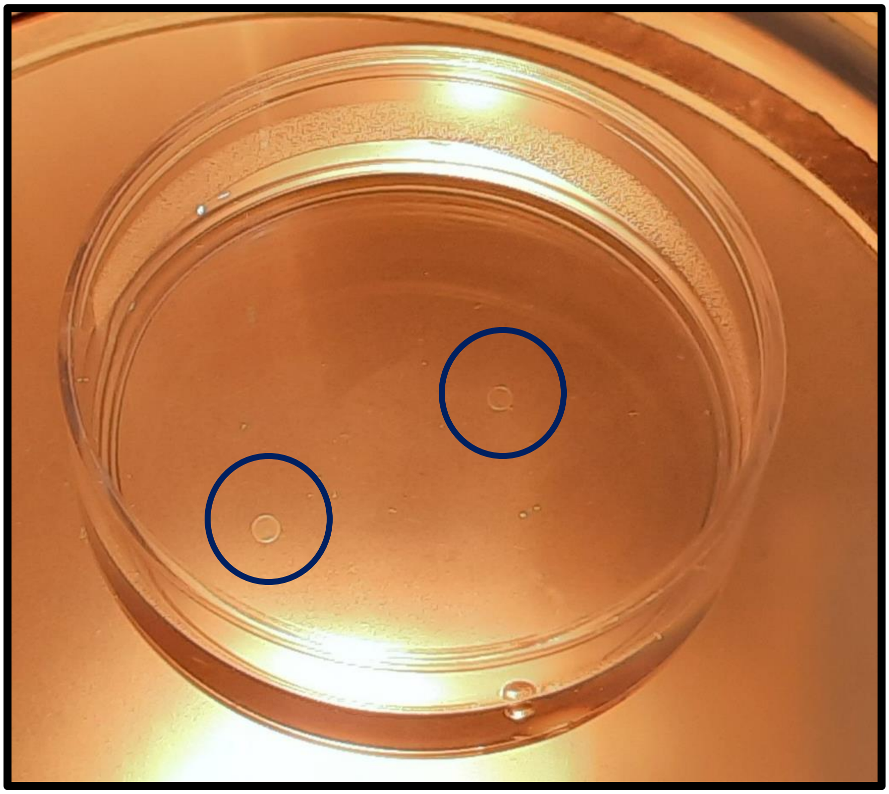

Two Day – 8 Mare Embryos (Visible with naked eye)

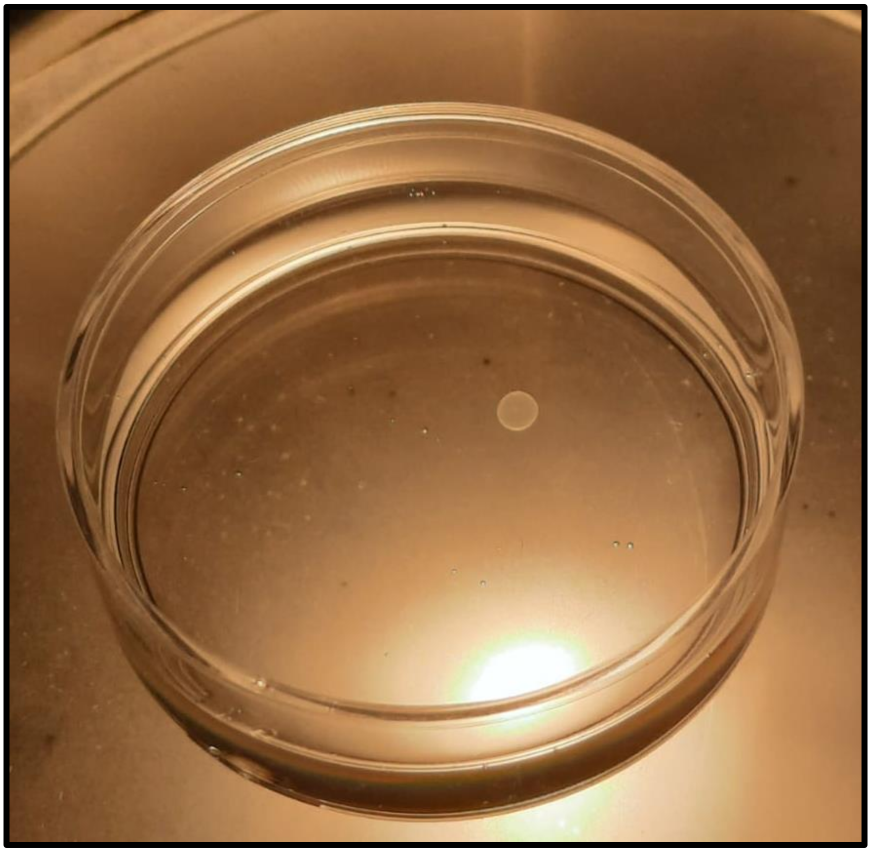

Day – 8 Mare Embryos (Visible with naked eye)

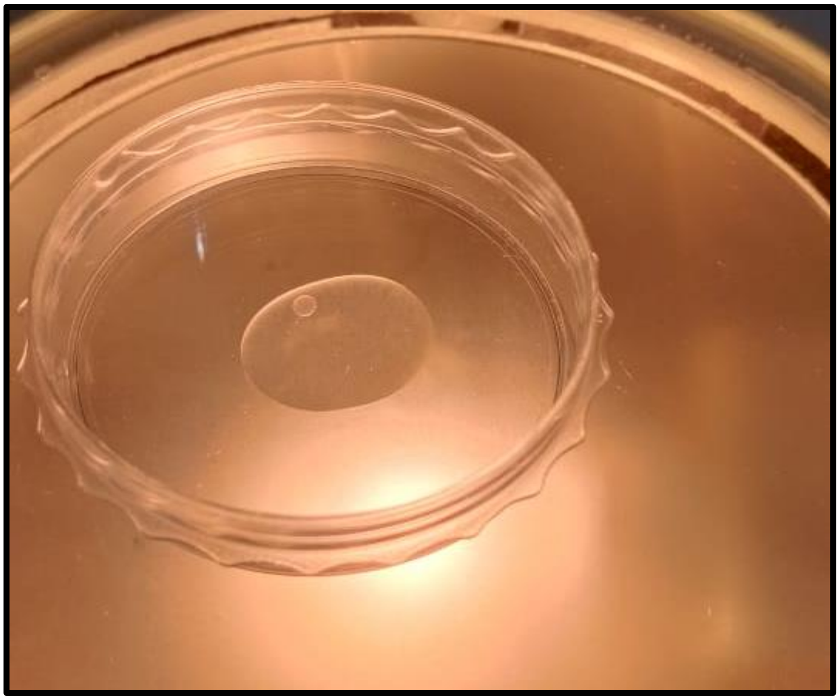

Day – 8 Mare Embryos (Visible with naked eye)

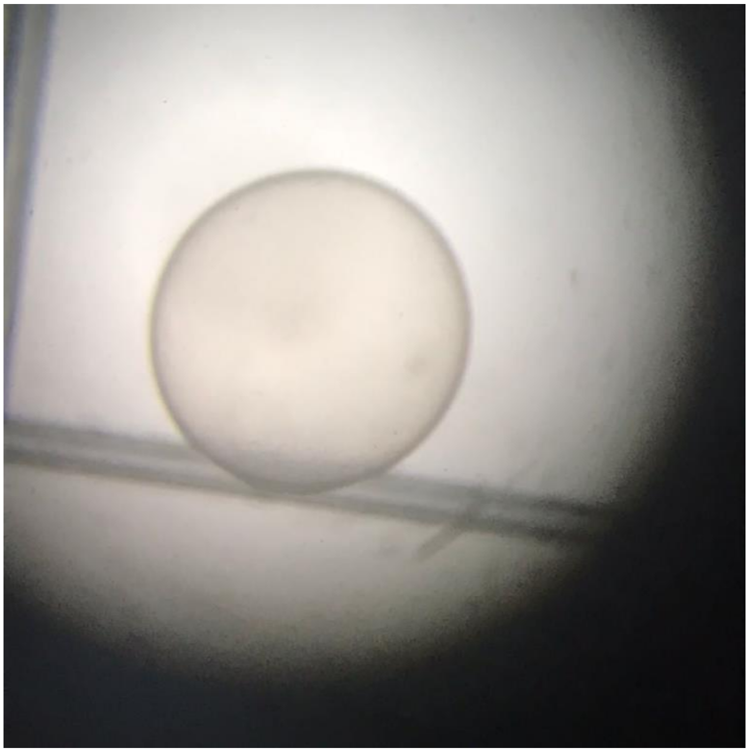

Embryo spotted in the embryo filter during searching

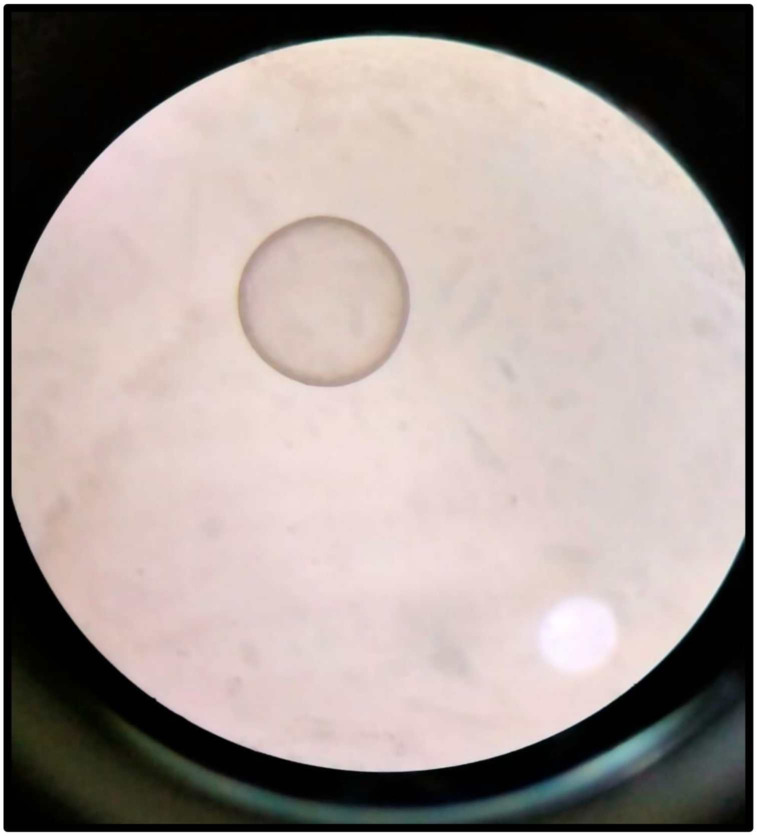

Expanded Blastocyst recovered from Donor Mare

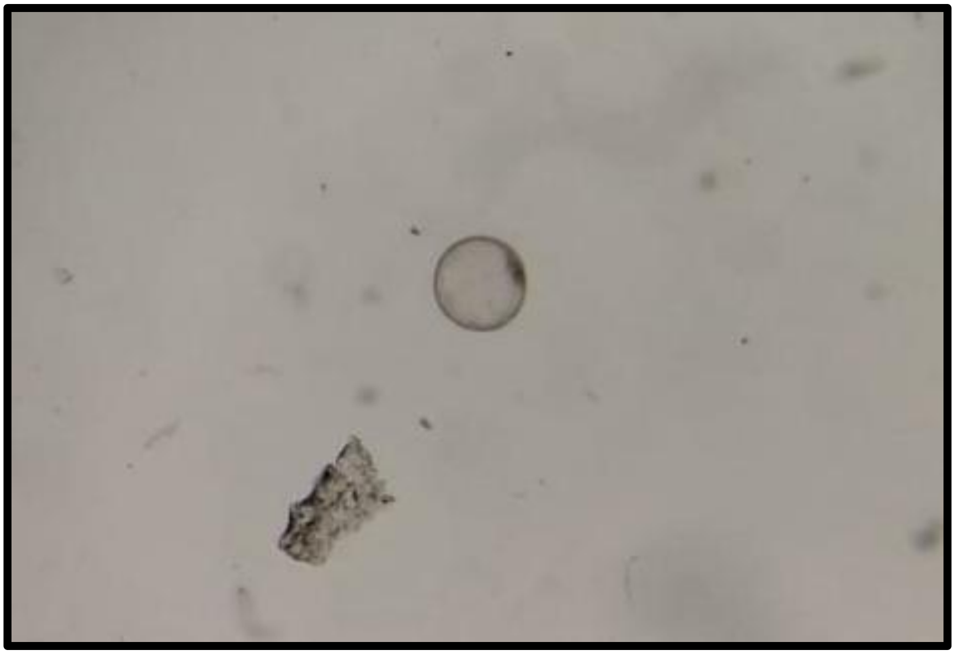

Blastocyst from an Argentino Polo Mare

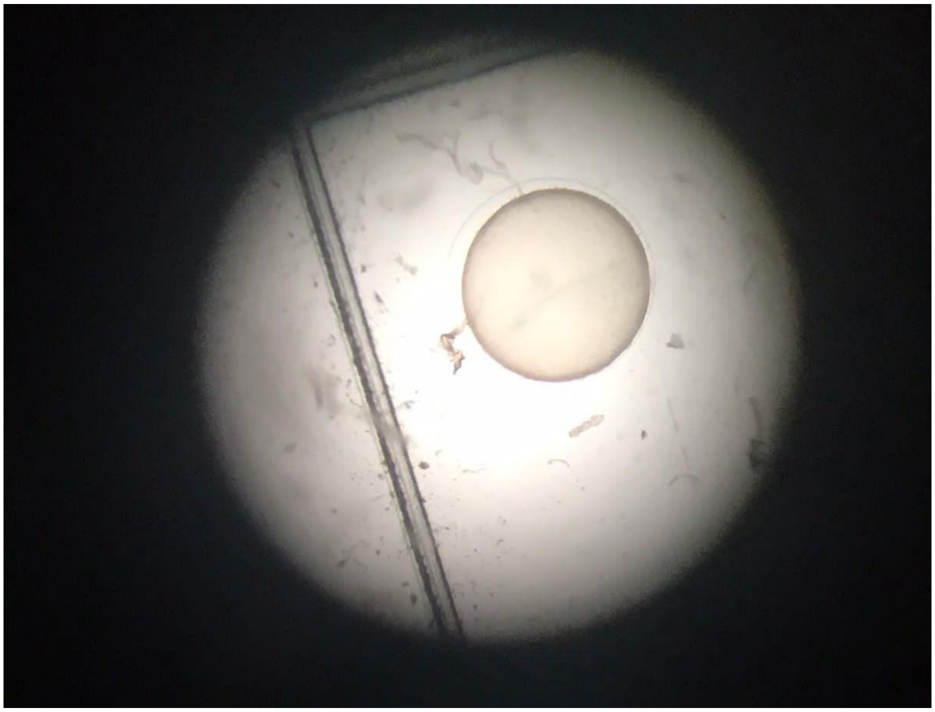

Expanded Blastocyst spotted in the embryo filter during searching
